# A Polyphenol-Enriched Supplement Exerts Potent Epigenetic-Protective Activity in a Cell-Based Model of Brain Ischemia

**DOI:** 10.3390/nu11020345

**Published:** 2019-02-06

**Authors:** Lara Faggi, Vanessa Porrini, Annamaria Lanzillotta, Marina Benarese, Mariana Mota, Dimitris Tsoukalas, Edoardo Parrella, Marina Pizzi

**Affiliations:** 1 Department of Molecular and Translational Medicine, University of Brescia, Viale Europa 11, 25123 Brescia, Italy; lfaggi87@gmail.com (L.F.); v.porrini@unibs.it (V.P.); annamaria.lanzillotta@unibs.it (A.L.); marina.benarese@unibs.it (M.B.); marianacmota@gmail.com (M.M.); marina.pizzi@unibs.it (M.P.); 2 European Institute of Nutritional Medicine, E.I.Nu.M., Viale Liegi 44, 00198 Rome, Italy; dr.tsoukalas@yahoo.com

**Keywords:** brain ischemia, OGD, NF-κB/RelA, HATs, HDACs, polyphenols, green tea, resveratrol

## Abstract

Bioactive components, due in part to their epigenetic properties, are beneficial for preventing several human diseases including cerebrovascular pathologies. However, no clear demonstration supports the idea that these molecules still conserve their epigenetic effects when acting at very low concentrations reproducing the brain levels achieved after oral administration of a micronutrient supplement. In the present study, we used a cellular model of brain ischemia to investigate the neuroprotective and epigenetic activities of a commercially available micronutrient mixture (polyphenol-enriched micronutrient mixture, PMM) enriched in polyphenols ((-)-epigallocatechin-3-gallate, quercetin, resveratrol), α-lipoic acid, vitamins, amino acids and other micronutrients. Mimicking the suggested dietary supplementation, primary cultures of mouse cortical neurons were pre-treated with PMM and then subjected to oxygen glucose deprivation (OGD). Pre-treatment with PMM amounts to provide bioactive components in the medium in the nanomolar range potently prevented neuronal cell death. The protection was associated with the deacetylation of the lysin 310 (K310) on NF-κB/RelA as well as the deacetylation of H3 histones at the promoter of *Bim*, a pro-apoptotic target of ac-RelA(K310) in brain ischemia. Epigenetic regulators known to shape the acetylation state of ac-RelA(K310) moiety are the histone acetyl transferase CBP/p300 and the class III histone deacetylase sirtuin-1. In view of that evidence, the protection we here report unveils the efficacy of bioactive components endowed with either inhibitory activity on CBP/p300 or stimulating activity on the AMP-activated protein kinase–sirtuin 1 pathway. Our results support a potential synergistic effect of micronutrients in the PMM, suggesting that the intake of a polyphenol-based micronutrient mixture can reduce neuronal vulnerability to stressful conditions at concentrations compatible with the predicted brain levels reached by a single constituent after an oral dose of PMM.

## 1. Introduction

Bioactive components have been extensively studied for their beneficial activities in preventing widespread human diseases, including cerebrovascular dysfunctions [1,2]. Bioactive components can modulate different epigenetic targets, including the histone acetylation/deacetylation balance, through the regulation of histone acetyltransferase (HAT) and histone deacetylase (HDAC) activities [3,4,5]. Acetylation of histones by HATs typically enhances transcription by reducing the affinity between DNA and histones, while the removal of acetyl groups by HDACs results in chromatin condensation, leading to the repression of gene transcription [6]. Notably, aberrant acetylation status resulting from the misregulation of HAT and/or HDAC activity has been associated with different pathologies, including brain ischemia [7].

Besides histones, HATs and HDACs can also modulate the acetylation state of a number of transcription factors, including NF-κB [8]. In the central nervous system, NF-κB plays a crucial role in regulating genes controlling either cell survival [9] or the apoptosis and inflammation associated with neurodegeneration [10]. We previously demonstrated that mechanisms affecting the acetylation state of the NF-κB/RelA subunit in brain ischemia can discriminate between protective and neurotoxic activation of the transcriptional factor [11]. Protective ischemic preconditioning and harmful ischemia induce similar levels of p50/RelA activation, but only the ischemic injury induces atypical RelA acetylation. The RelA that translocates to the nucleus in primary cortical neurons exposed to preconditioning oxygen and glucose deprivation (OGD) or in cortices of mice subjected to preconditioning middle cerebral artery occlusion (MCAO) shows a general lysine deacetylation. Conversely, as demonstrated by mutagenesis analysis, the RelA activated in neurons exposed to lethal OGD or in the cortices of mice subjected to noxious ischemia displays a general lysine deacetylation, but a site-specific acetylation at the lysine 310 (K310) residue [12,13]. The aberrant RelA acetylation in neurons subjected to OGD was associated with reduced histone acetylation and with the transcription of the pro-apoptotic *Bim* gene, as indicated by increased H3 acetylation at the *Bim* promoter [14,15,16]. The aberrantly acetylated RelA co-immunoprecipitated with the HAT p300/CBP, suggesting a role of p300/CBP in K310 acetylation during the lethal exposure of neuronal cells to OGD [12].

In the present work, we investigated the neuroprotective activity of a polyphenol-enriched micronutrient mixture (PMM), containing (-)-epigallocatechin-3-gallate (EGCG), quercetin, resveratrol, α-lipoic acid (LA), vitamins, amino acids and other micronutrients in primary mouse cortical neurons exposed to OGD. In addition, we studied the epigenetic mechanisms involved in the activity of the bioactive components mixture by focusing on the acetylation state of either HAT or HDAC target proteins.

## 2. Materials and Methods

### 2.1. PMM Composition

In this study, we tested the neuroprotective effect of the antioxidant supplements contained in MyAntiOxidant (MA) and the multivitamin supplements contained in MyHealth (MH) (Meetab Srl, Milan, Italy). MA and MH were generously gifted by Meetab Srl. The daily recommended dose of the supplements is 4 tablets of MA (https://www.meetab.it/en/23-antiossidante.html) and 1 tablet of MH (https://www.meetab.it/en/22-my-health.html). The supplement powders, in accordance with the manufacturer’s recommendations, were mixed at the ratio MA:MH = 4:1mg to obtain the PMM. Table 1 shows the composition of the combined multivitamin and antioxidant supplement. The compositions of MA and MH have been certified by the manufacturer in terms of the quality and concentration of raw materials, the exclusion of GMO (genetically modified organism), radiation procedure and contaminants listed in the Annex II regulations (EU) No 1169/2011 (http://meetab.it/certificates/CoAs_My-AntiOxidant.pdf; https://www.meetab.it/certificates/CoAs_My-Health.pdf; see also supplementary file). Briefly, a daily dose of MA and MH supplements contains 850.00 mg of polyphenols and LA, 1272.66 mg of vitamins, 1222.50 mg of amino acids, 454.00 mg of fruit and plant extracts and 140.63 mg of mineral salts.

### 2.2. PMM Preparation

Based on literature data, we estimated that the possible neuroprotective effect of the PMM against anoxic insults could be mediated by the presence of green tea in the supplement and, in particular, of EGCG, the polyphenol present in the greatest quantity in the green tea [17,18,19,20,21,22], together with other bioactive components present in large quantities in the mixture. The PMM concentrations tested in the present study are reported in Table 2 with the corresponding contents of main bioactive components. PMM was dissolved in dimethyl sulfoxide (DMSO) and diluted before application to a final DMSO concentration lower than 0.3%. 

### 2.3. Cell Culture

Primary cultures of mouse cortical neurons were prepared from 15-day-old embryonic C57Bl/6 mice (Charles River, Calco, Italy) and maintained in culture as previously described [23,24]. All animal studies were approved by the Animal Research Committees of the University of Brescia and followed Directive 2010/63/EU of the European Parliament and of the Council of 22 September 2010 on the protection of animals used for scientific purposes. Cells were plated at a density of 2.0 × 10^6^ cells in 21 cm^2^ culture dishes (Nunc, Germany) for Western blot (WB) analyses, 2.2 × 10^5^ cells in 2 cm^2^ culture dishes for the viability studies and 9 × 10^6^ cells in 56 cm^2^ culture dishes for the chromatin-immunoprecipitation (ChIP) assay.

### 2.4. OGD and Measurement of Lactate Dehydrogenase (LDH) Release

Primary cortical neurons were exposed from day 7 in vitro (DIV) to different concentrations of PMM ranging from 0.00884 to 26.5 pg/mL (corresponding to the following contents of the main bioactive components: EGCG 0.0010–3.0 nM; quercetin 0.0011–3.3 nM; LA 0.0016–4.9 nM; resveratrol 0.0005–1.5 nM; vitamin C 0.0089–26.8 nM; vitamin E 0.0006–1.9 nM; vitamin B6 0.0002–0.5 nM; N-acetyl L-cysteine 0.0015–4.5 nM). The treatments were renewed at every change of media. The same amount of DMSO contained in the PMM was used as a vehicle.

OGD was carried out at DIV 11, as previously described [23,24]. Briefly, cells were incubated with deoxygenated glucose-free balanced salt solution and transferred to an air-tight chamber fluxed with 95% N_2_ and 5% CO_2_ for 10 min to reach an oxygen concentration lower than 0.4%. Cortical neurons were exposed to OGD for 3 h. A parallel set of cultured neurons were incubated for 3 h with a normal oxygenated, balanced salt-solution containing glucose and used as a control. After the anoxic incubation, neurobasal medium containing 0.4% B27 supplement was added to cortical neurons for 24 h to allow recovery under normoxic conditions. At the end of the recovery period, we estimated the neuronal injury by assessing the ratio between the amount of lactate dehydrogenase (LDH) released in culture medium and the total releasable LDH using the CytoTox 96^®^ Non-Radioactive Cytotoxicity Assay (Promega, Fitchburg, WI, USA). All the experiments were run in triplicate. 

### 2.5. WB and Co-Immunoprecipitation (co-IP) Assays

Nuclear protein extracts were prepared as previously described [16] from primary cortical neurons exposed to 3 h OGD and 2 h recovery in culture medium. WB analyses and co-IP studies were carried out as previously described [16]. For WB analyses, nuclear proteins (20 μg) were resolved by Bolt™ 4–12% Bis-Tris Plus SDS/polyacrylamide pre-cast gels (Invitrogen, Carlsbad, CA, USA). Immunodetection was performed by incubating the membrane overnight at 4 °C with the following primary antibodies: rabbit anti-Acetyl H3 (K9-18) (#07-593, Upstate-Millipore, Burlington, MA, USA), rabbit anti-Acetyl H4 (K16) (#06-762, Upstate-Millipore), rabbit anti-H3 (#9715, Cell Signaling Technology, Danvers, MA, USA) and rabbit anti-H4 (#07-108, Upstate-Millipore). For co-IP analyses, 40 µg of nuclear extracts were incubated at 4 °C overnight with 2 μg of goat anti-RelA antibody (#sc-372GX, Santa Cruz Biotechnology, Dallas, TX, USA), and co-immunoprecipitated proteins were detected by WB using the following antibodies: rabbit anti-Acetyl-RelA (K310) (#3045, Cell Signaling Technology), rabbit anti-Acetyl-K (#06-933, Upstate-Millipore) and rabbit anti-RelA (#sc-372, Santa Cruz Biotechnology). Quantification of protein expression was performed by densitometric analysis of immunoblots using Gel Pro.3 analysis software (MediaCybernetics, Rockville, MD, USA).

### 2.6. ChIP Assay

ChIP assays were performed in primary cortical neurons, as previously described [16]. To study H3 histone acetylation at the *Bim* promoter in neuronal cultures exposed to 3 h OGD and then 2 h recovery in culture medium, we used a ChIP assay kit (#9003S, Cell Signaling Technology). The sheared chromatin was incubated with anti-acetyl H3 (K9/18) or anti-IgG (negative control) overnight at 4 °C. An aliquot of chromatin, not incubated with antibody, was used as the input control sample. Antibody-bound protein/DNA complexes were washed, eluted, treated with proteinase digest proteins and subjected to real-time polymerase chain reaction (qRT-PCR) analyses. The specific primers used to amplify the mouse *Bim* promoter were as follows:

forward, 5-CTGGATGCAGGTTGGGTAG-3
reverse, 5 GGGAATGAGAAAGTTAGCTGGA-3.

These specific primers generated a 410-bp product. Incorporation of the SYBR Green dye into the PCR products was monitored in real-time with a ViiA™ 7 Real-Time PCR detection system (Applied Biosystems, Bedford, MA, USA), allowing the determination of the threshold cycle (C_T_) at which the exponential amplification of PCR products began. C_T_ values were normalized over corresponding C_T_ values obtained by IgG immunoprecipitation and further normalized over relative C_T_ values obtained in input (no antibody) chromatin. Final data obtained in neurons exposed to OGD (with or without pretreatment with PMM) were then normalized to data obtained in control neurons. 

### 2.7. Statistical Analysis

Data are expressed as mean ± standard error, and the statistical significance of differences between groups was evaluated by one-way ANOVA followed by Dunnett’s multiple comparison test using GraphPad Prism 5 software (GraphPad Software, Inc., San Diego, CA, USA) *p* < 0.05 was considered to be significant.

## 3. Results

### 3.1. Pre-Treatment with PMM Elicits Neuroprotection in Cortical Neurons Exposed to OGD

**Figure 1:**
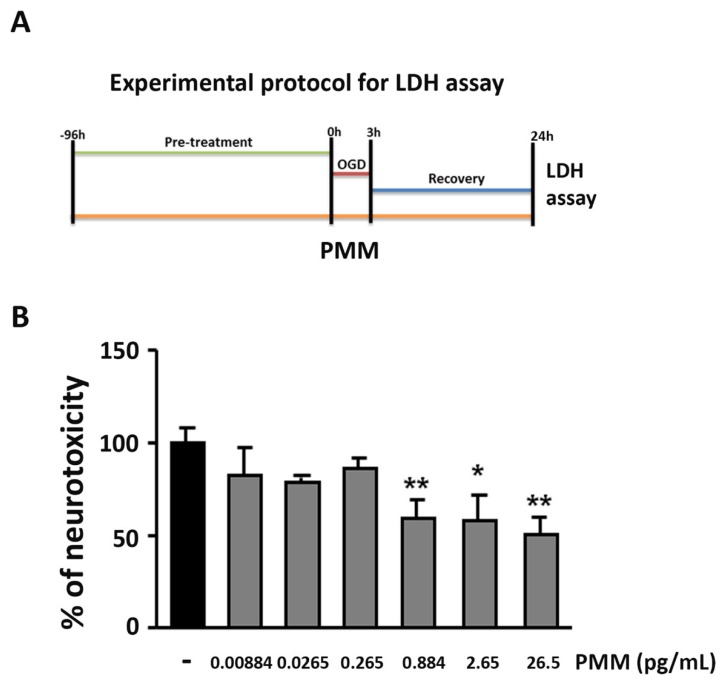
**Pre-treatment with the polyphenol-enriched micronutrient mixture (PMM) promotes neuroprotection in cortical neurons exposed to oxygen and glucose deprivation (OGD).** (**A**) Experimental protocol for lactate dehydrogenase (LDH) toxicity assay in cortical neurons. Cells were pre-treated with the PMM at day in vitro (DIV) 7. At DIV 11, cells were exposed to 3 h of OGD and then maintained in recovery medium for 24 h. The mixture was renewed at every change of media. (**B**) LDH assay was performed at the end of the 24 h of recovery. Different doses of PMM were used in cells. DMSO was used as vehicle in control cells (neurons exposed to OGD). The neurotoxicity of the PMM-treated neurons is represented as a percentage relative to the neurotoxicity in the vehicle-treated neurons (OGD-exposed neurons), arbitrarily set at 100%. The effective neuroprotective doses of the mixture were 0.884, 2.65 and 26.5 pg/mL, which corresponded to the following doses of the main bioactive components of the mixture: (-)-epigallocatechin-3-gallate (EGCG) 0.10–3.0 nM; quercetin 0.11–3.3 nM; α-lipoic acid (LA) 0.16–4.9 nM; resveratrol 0.05–1.5 nM; vitamin C 0.89–26.8 nM; vitamin E 0.06–1.9 nM; vitamin B6 0.02–0.5 nM; N-acetyl L-cysteine 0.15–4.5 nM. The bar graph represents the mean of three different experiments. * *p* < 0.05 and ** *p* < 0.01 vs. OGD (one-way ANOVA followed by Dunnett’s multiple comparison test).

We previously showed that in primary cultures of mouse cortical neurons exposed to OGD, apoptosis precedes necrosis. This was indicated by the early TUNEL-positivity displayed by the cells within 6 h after the OGD and the parallel release of cytochrome c in the cytosol in the absence of LDH release. Subsequent necrosis causes progressive elevation of the extracellular LDH level that becomes clearly detectable in the culture medium 24 h after the OGD exposure [15,25]. Starting from the DIV 7, PMM or vehicle were added to the culture medium of cortical neurons and maintained until the end of the OGD recovery (DIV 12). At DIV 11, cortical neurons were exposed to 3 h OGD followed by 24 h of recovery (Figure 1A—experimental protocol). The treatment with PMM at concentrations ranging from 0.884 to 26.5 pg/mL reduced cell death by 40% (Figure 1B). 

### 3.2. Pre-Treatment with PMM Prevents the Derangement of RelA Acetylation in Cortical Neurons Subjected to OGD

Previous studies demonstrated that, in neuronal cultures exposed to OGD, the anti-apoptotic or pro-apoptotic activity of NF-κB/p50-RelA relies on a different acetylation state of the RelA subunit, the latter depending on the duration of OGD exposure. While a “protective”, preconditioning OGD (1 h) promotes a general deacetylation of the RelA lysine residues, the lethal OGD (3 h) promotes the deacetylation of RelA lysines with the exception of the 310 residue [10,12,13,16].

**Figure 2:**
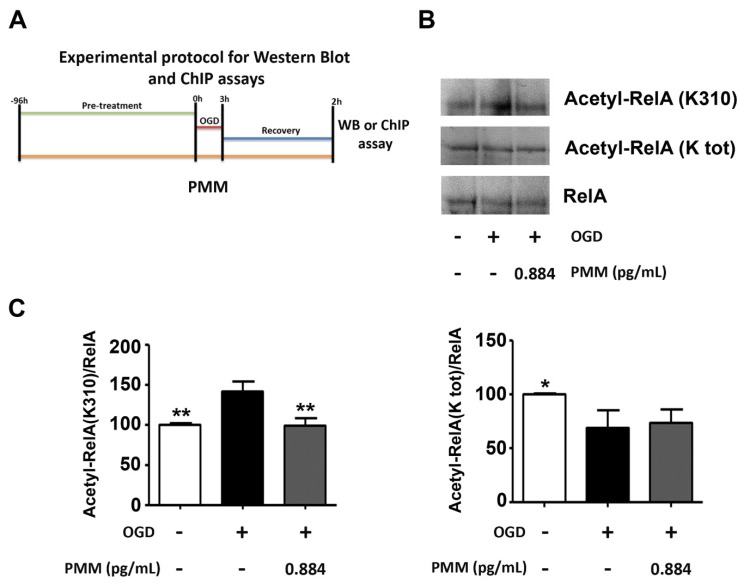
**Pre-treatment with PMM reduces ac-RelA(K310) acetylation in cortical neurons exposed to OGD.** (**A**) Experimental protocol for the Chromatin immunoprecipitation (ChIP) assay and Western blot (WB) assay in cortical neurons. Cells were pre-treated with the PMM at DIV 7. At DIV 11, cells were exposed to 3 h of OGD and then maintained in recovery medium for 2 h. The mixture was renewed at every change of media. ChIP assays or WB analyses were performed at the end of the 2 h of recovery. The lowest neuroprotective dose of the PMM was added to cells (EGCG 0.10 nM; quercetin 0.11 nM; LA 0.16 nM; resveratrol 0.05 nM; vitamin C 0.89 nM; vitamin E 0.06 nM; vitamin B6 0.02 nM; N-acetyl L-cysteine 0.15 nM). DMSO was used as vehicle in control cells and in neurons exposed to OGD. (**B**,**C**) Co-IP assay of RelA acetylation in cortical neurons exposed to OGD. The acetylation levels of total lysines (K) of RelA were unchanged after the treatment with the mixture when compared to OGD-treated cells, while the treatment with the PMM restored the normal RelA(K310) acetylation. The bar graphs represent the mean of three different experiments. * *p* < 0.05 and ** *p* < 0.01 vs. OGD-treated cells (one-way ANOVA followed by Dunnett’s multiple comparison test).

In order to investigate the molecular mechanism associated with the neuroprotection, we evaluated the capability of the lowest neuroprotective dose of the mixture to revert the derangement of RelA acetylation. PMM at 0.884 pg/mL or vehicle were added to the cultured cortical neurons at DIV 7 and maintained until 2 h after the OGD period. At the end, sister cultures were processed for the separation of nuclear proteins or ChIP assay. Figure 2A shows the experimental protocol applied for the ChIP, WB and co-IP analyses. Co-IP assays in nuclear extracts of cortical neurons exposed to 3 h OGD and 2 h recovery showed the ability of PMM to limit the RelA(K310) acetylation without improving the general RelA deacetylation. As reported for the OGD precondition setting [10,12], the ac-RelA(K310)/RelA ratio decreased, while the general ac-RelA/RelA remained lower than that in control conditions (Figure 2B,C).

### 3.3. Pre-Treatment with PMM Reduces H3 Histone Acetylation at the *Bim* Promoter in Neurons Exposed to OGD

**Figure 3:**
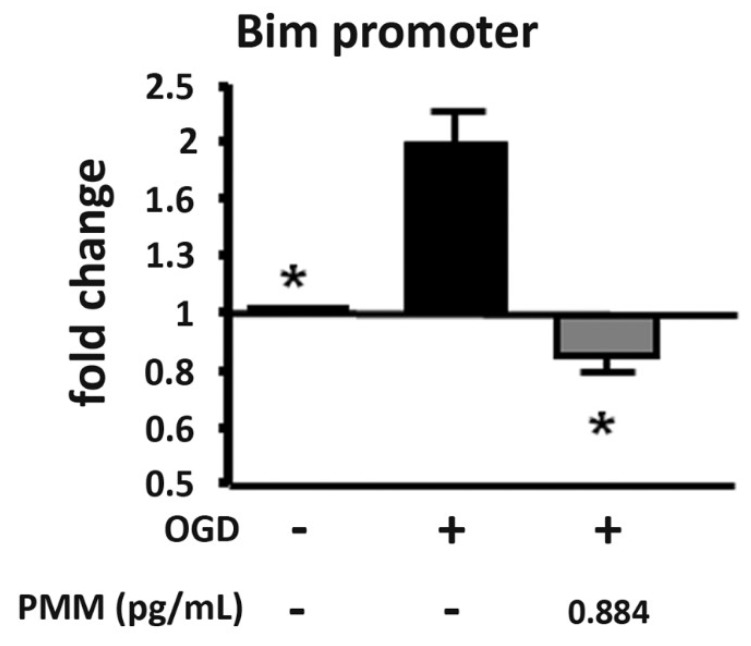
**Pre-treatment with PMM reduces H3 histone acetylation at the *Bim* promoter in neurons subjected to OGD.** ChIP assay in cortical neurons exposed to OGD. Cells were pre-treated with the PMM at DIV 7. At DIV 11, cells were exposed to 3 h of OGD and then maintained in recovery medium for 2 h. The mixture was renewed at every change of media. The ChIP assay was performed at the end of the 2 h of recovery. The lowest neuroprotective dose of the PMM was added to cells (EGCG 0.10 nM; quercetin 0.11 nM; LA 0.16 nM; resveratrol 0.05 nM; vitamin C 0.89 nM; vitamin E 0.06 nM; vitamin B6 0.02 nM; N-acetyl L-cysteine 0.15 nM). DMSO was used as vehicle in control cells and in neurons exposed to OGD. The ChIP assay revealed an epigenetic effect of the mixture in reducing the acetylation state of histones H3 on the promoter of the pro-apoptotic gene *Bim*. The bar graph represents the mean of three different experiments. * *p* < 0.05 vs. OGD-treated cells (one-way ANOVA followed by Dunnett’s multiple comparison test).

As a marker of the epigenetic regulation of apoptosis, which precedes necrosis in neurons exposed to OGD, we investigated the histone acetylation at the promoter of the pro-apoptotic gene *Bim* [14], specifically targeted by the ac-RelA(K310) [12,16]. In line with previous data, the ChIP assay demonstrated a specific increase of H3 acetylation (K9/18) at the *Bim* promoter in neurons exposed to OGD [12,14,16]. The histone acetylation decreased in cells treated with the lowest neuroprotective dose of the mixture (PMM at 0.884 pg/mL) (Figure 3).

### 3.4. Pre-Treatment with PMM Does Not Restore the Histone Acetylation Status in Cortical Neurons Subjected to OGD

**Figure 4:**
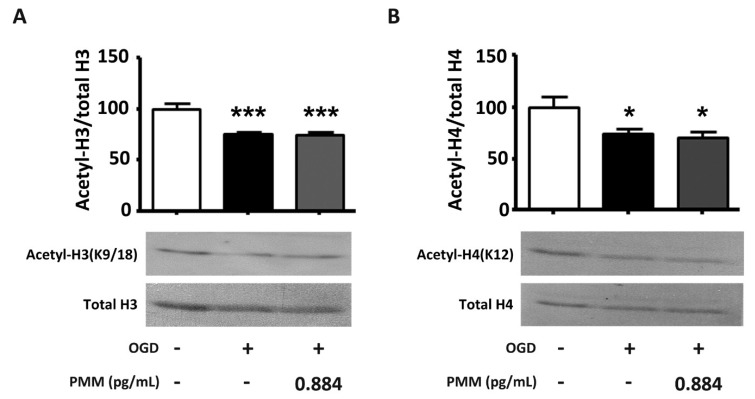
**Pre-treatment with PMM does not modulate the acetylation of histones H3 and H4 in cortical neurons subjected to OGD.** WB of acetylation levels of H3 (**A**) and H4 (**B**) in cortical neurons exposed to OGD. Cells were pre-treated with the PMM at DIV 7. At DIV 11, cells were exposed to 3 h of OGD and then maintained in recovery medium for 2 h. The mixture was renewed at every change of media. The WB assay was performed at the end of the 2 h of recovery. The lowest neuroprotective dose of the PMM was used (EGCG 0.10 nM; quercetin 0.11 nM; LA 0.16 nM; resveratrol 0.05 nM; vitamin C 0.89 nM; vitamin E 0.06 nM; vitamin B6 0.02 nM; N-acetyl L-cysteine 0.15 nM). DMSO was used as a vehicle in control cells and neurons exposed to OGD. The acetylation levels of histones H3 and H4 did not return to control levels after treatment with the mixture. The bar graph represents the mean of three different experiments. * *p* <0.05 and *** *p* < 0.001 vs. OGD-treated cells (one-way ANOVA followed by Dunnett’s multiple comparison test).

We then evaluated the effect of PMM in modulating the acetylation state of histones in cortical neurons subjected to OGD. The global H3 (K9–18) and H4 (K16) acetylation, measured by the WB analyses of nuclear extracts, appeared reduced, in line with previous evidence obtained either in cells exposed to OGD or in in vivo brain ischemia [16,26]. However, the general histone deacetylation was not reversed by the PMM application at the lowest neuroprotective dose (0.884 pg/mL) (Figure 4A and 4B), thus excluding a possible general inhibitory effect of PMM on HDAC activity. 

## 4. Discussion

Here, we demonstrated the neuroprotective effect of a polyphenol-enriched nutrient supplement in a cell-based model of ischemic stroke involving the primary cortical neurons exposed to lethal OGD. 

**Figure 5:**
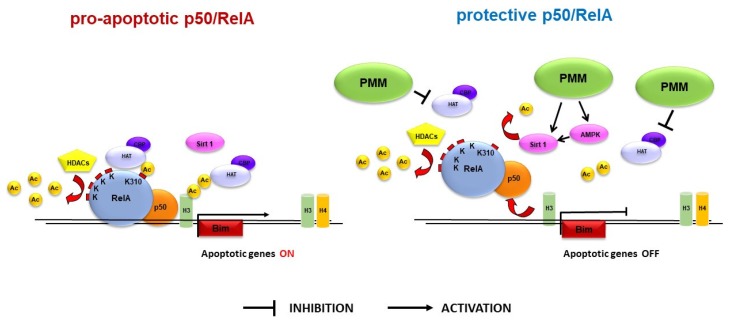
**PMM-mediated defense from ischemic injury.** During OGD, the activated RelA within the p50/RelA dimer is in its aberrantly acetylated form, consisting of a hypoacetylated state with the exception of the residue K310. In this condition, p50/RelA binds the pro-apoptotic *Bim* promoter, an event followed by specific histone H3 acetylation at the target gene promoter. We demonstrated that the pre-treatment of neurons with PMM was able to limit the RelA(K310) acetylation and the H3 acetylation at the *Bim* promoter. Neither the general acetylation of RelA, nor the general histone acetylation, were restored by the supplement mixture, suggesting that PMM acts as a histone acetyltransferase (HAT) inhibitor, rather than a histone deacetylase (HDAC) inhibitor. We speculate that the correction of the aberrant RelA acetylation in K310 could be promoted by components of the mixture able to act as HAT inhibitors, including EGCG, quercetin and LA. Also, the activation of the AMPK–sirtuin 1 pathway by EGCG, quercetin, LA, vitamin C, vitamin E, vitamin B6 and N-acetyl-L-cysteine (NAC) could contribute to the ac-RelA(K310) deacetylation.

Neuroprotection was associated with changes in the acetylation state of NF-κB/RelA. In our experimental setting, we confirmed the aberrant acetylation of RelA after the lethal OGD exposure. The PMM treatment was able to reduce the ac-RelA(K310) acetylation without improving the deacetylation of the other lysine residues. It can be inferred that PMM could induce a switch of RelA from the “lethal pro-apoptotic” form to the “preconditioning protective” form [12]. By undergoing the aberrant acetylation, RelA binds the pro-apoptotic *Bim* promoter [16], an event followed by specific histone acetylation at the target gene promoter. The increased H3 acetylation at the *Bim* promoter is an early marker of the triggered apoptotic process [16]. In the present work, we demonstrated that the treatment of cells with PMM was able to limit the H3 acetylation at the *Bim* promoter, suggesting that the mixture of bioactive components could regulate the expression of this pro-apoptotic gene (Figure 5). 

In addition to changing the acetylation state of RelA, lethal ischemia produces a significant reduction of H3 and H4 histone acetylation [16,26]. Our data confirmed the general reduction of H3 and H4 histone acetylation after OGD exposure. In contrast to the effect produced by HDAC inhibitors in models of brain ischemia [16,27,28], PMM did not restore the general histone acetylation. This suggests that the supplement mixture acts as a HAT inhibitor, rather than a HDAC inhibitor. 

EGCG, one of the main components of the mixture, has been proposed to act as either an HDAC inhibitor or a HAT inhibitor [29,30,31,32]. In accordance with the hypothesis that in our model EGCG could work as a HAT inhibitor, Choi and colleagues reported that EGCG reduced ac-RelA(K310) acetylation by directly inhibiting the activity of HAT enzymes, p300 and CPB [29]. However, it cannot be excluded that, at concentrations higher than those used in our experimental setting, the EGCG inhibition of HAT can lead to the reduction of histone acetylation at the HDAC promoter, resulting in the inhibition of HDAC expression. Several bioactive components of the mixture could act as HAT inhibitors, thus contributing to the observed neuroprotective effect. Besides EGCG, among the compounds present at higher concentrations, quercetin and LA inhibit p300/CBP [33,34] and have been reported to exert neuroprotective effects in cellular and animal models of brain ischemia [17,18,19,20,21,35,36,37,38,39,40,41]. Therefore, one of the possible mechanisms involved in the RelA regulation could very well be the inhibition of p300/CBP by PMM (Figure 5).

Recently, we showed that the combination of MS-275 or valproate, two class-I HDAC inhibitors, with resveratrol, an activator of the AMP-activated protein kinase (AMPK)–sirtuin 1 pathway, could restore normal RelA acetylation and elicit neuroprotection in cortical neurons exposed to OGD and in animal models of brain ischemia [16,27] or amyotrophic lateral sclerosis (ALS) [42]. In light of these findings, we can speculate that the resveratrol present in the PMM, by activating the AMPK-sirtuin 1 pathway, could participate in ac-RelA(K310) deacetylation and work synergistically with other compounds to limit NF-κB-mediated apoptosis [12,16,43] (Figure 5). Recent findings indicate that also other molecules of the mixture, including EGCG, quercetin, LA, vitamin C, vitamin E, vitamin B6 and N-acetyl-L-cysteine (NAC) are able to activate the AMPK–sirtuin 1 pathway [44,45,46,47,48,49,50,51,52,53,54,55], suggesting that these compounds could amplify the action of resveratrol. Future experiments will investigate the effects of sirtuin-1 and AMPK inhibitors in blocking the neuroprotective activity of PMM.

The broccoli extract present in the PMM could also contribute to the observed neuroprotective effect. The isothiocyanate compound sulforaphane (1-isothiocyanato-4-methylsulfinylbutane), which is highly produced in a variety of cruciferous vegetables (*Brassica*; e.g., broccoli), has been found to protect hippocampal neurons against OGD at a concentration of 0.5 μM [56] and to inhibit HDAC activity in mouse cortical neurons at 10–20 μM [57]. However, the lack of HDAC inhibitor-like effects of PMM, i.e., the failure to increase the general acetylation of RelA and H3 (K9–18) and H4 (K16) histones [16], might very well exclude the participation of sulforaphane to the synergistic effects of the other compounds in modulating RelA acetylation.

In our experimental setting, the expected concentrations of the compounds present in the mixture were thousands-fold lower than those found to be protective when individual compounds were tested in neurons exposed to OGD [16,18,35,41,58,59,60,61,62]. Similarly, the compounds’ concentrations in the PMM were remarkably lower than those able to inhibit HATs or activate the AMPK–sirtuin 1 pathway when the bioactive components were tested individually [16,29,33,34,44,45,46,47,48,49,51]. The neuroprotective effect and the epigenetic activity displayed by these molecules, despite their low concentrations, indicate that a synergistic interaction between different bioactive components may occur in the PMM. Notably, the beneficial effect occurs at concentrations in the nanomolar range, compatible with the predicted brain levels of most of the bioactive components reachable at the recommended daily assumption dose of PMM [63,64,65,66,67,68,69,70,71]. Although the approach to studying PMM has the limitation of not discriminating the role of each specific compound separately, it offers the valuable advantage of evaluating the final result of multiple nutritional supplements when administered in combination.

## 5. Conclusions

In conclusion, we demonstrated that a nutrient combination of antioxidant and multivitamin supplements containing polyphenols could shape the acetylation of RelA and reduce the neuronal vulnerability to conditions associated with oxygen and glucose deprivation. The protective activity of PMM pretreatment suggests the beneficial effect of this supplementation in increasing resilience to brain damage derived from potential ischemic events in treated subjects. 

## Figures and Tables

**Table 1:** Polyphenol-enriched micronutrient mixture (PMM) composition.

PMM Components	Amount per Daily Dose (mg)	Content per 100 mg (%)
**Polyphenols and α-lipoic acid**	**850.00**	**21.57**
green tea extract (40% EGCG)	500.00 (200.00)	12.69 (5.08)
quercetin	150.00	3.81
α-lipoic acid	150.00	3.81
resveratrol	50.00	1.27
**Vitamins**	**1272.66**	**32.30**
vitamin C (*)	890.00	22.59
vitamin E (**)	120.00	3.05
vitamin B3 (***)	70.00	1.78
vitamin B1	50.00	1.27
vitamin B6 (****)	18.00	0.46
β-carotene	14.00	0.36
others	110.66	2.81
**Amino acids**	**1222.50**	**31.03**
L-lysine	525.00	13.33
L-glutamine	150.00	3.81
L-proline	120.00	3.05
N-acetyl L-cysteine	110.00	2.79
L-arginine	105.00	2.67
L-methionine	105.00	2.67
glycine	60.00	1.52
others	47.50	1.21
**Fruits and Plants extract**	**454.00**	**11.52**
broccoli extract	150.00	3.81
others	304.00	7.72
**Mineral salts**	**140.63**	**3.57**
calcium (*****)	50.00	1.27
potassium (citrate)	30.00	0.76
zinc (amino acid chelated)	12.50	0.32
magnesium (citrate)	10.00	0.25
manganese (gluconate)	8.00	0.20
phosphorus (******)	8.00	0.20
others	22.12	0.56
**Total**	**3939.79**	**100.00**

(*) 550 mg ascorbic acid / 300 mg calcium ascorbate / 40 mg ascorbyl palmitate; (**) 80 mg α-tocopherol / 20 mg mixed α-, β-, γ-, δ- tocopherols/20 mg mixed tocotrienols; (***) 60 mg nicotinamide / 10 mg nicotinic acid (****) as pyridoxal 5′-phosphate; (*****) derived from 40 mg calcium carbonate and 10 mg calcium phosphate; (******) derived from calcium phosphate.

**Table 2:** PMM concentrations and corresponding contents of the main bioactive components.

Nutrients	Concentrations					
**PMM (pg/mL)**	0.00884	0.0265	0.265	0.884	2.65	26.5
**EGCG (nM)**	0.0010	0.0030	0.030	0.10	0.30	3.0
**quercetin (nM)**	0.0011	0.0033	0.033	0.11	0.33	3.3
**α-lipoic acid (nM)**	0.0016	0.0049	0.049	0.16	0.49	4.9
**resveratrol (nM)**	0.0005	0.0015	0.015	0.05	0.15	1.5
**vitamin C (nM)**	0.0089	0.0268	0.268	0.89	2.68	26.8
**vitamin E (nM)**	0.0006	0.0019	0.019	0.06	0.19	1.9
**vitamin B6 (nM)**	0.0002	0.0005	0.005	0.02	0.05	0.5
**N-acetyl L-cysteine (nM)**	0.0015	0.0045	0.045	0.15	0.45	4.5

## Supplementary Materials

The following is available at http://www.mdpi.com/2072-6643/11/2/345/s1, The manufacturer certificate of analysis for the drug complexes, with the specific datasheet of the most relevant components of the mixture, as focused in Table 2 (EGCG in the green tea, quercetin, alpha-lipoic acid, resveratrol, vitamin C, vitamin E, vitamin B6 and N-acetyl L-cysteine).

## Abbreviations

AMPK, AMP-activated protein kinase; ChIP, chromatin immunoprecipitation; DIV, day in vitro; DMSO, dimethyl sulfoxide; EGCG, (-)-epigallocatechin-3-gallate; GMO, genetically modified organism; HAT, histone acetyltransferase; HDAC, histone deacetylase; LA, α-lipoic acid; LDH, lactate dehydrogenase; MCAO, middle cerebral artery occlusion; MA, MyAntiOxidant; MH, MyHealth; NAC, N-acetyl-L-cysteine; OGD, oxygen glucose deprivation; PMM, polyphenol-enriched micronutrient mixture.
