# Supplementary material for: A Polyphenol-Enriched Supplement Exerts Potent Epigenetic-Protective Activity in a Cell-Based Model of Brain Ischemia
Source: Nutrients. 2019 Feb 6;11(2):345. doi: 10.3390/nu11020345 (PMC6412333; doi:10.3390/nu11020345)
Supplement: Supplementary file 1 [file nutrients-11-00345-s001.pdf]

MEETAB CERTIFICATE OF ANALYSIS

Components of products provided to the University of Brescia:

- MY HEALTH MEETAB
- MY ANTIOXIDANT MEETAB

Products Formula: **Meetab s.r.l.**
Contractor: Naturfarma s.r.l..

Raw materials analysis performed by Naturfarma s.r.l. (contractor)

NATUR-FARMA s.r.l. is authorized to produce and package supplements by manufacturability approval No. CE IT 027 VR 00025 – registration/authorization: 050ND03082 (Pressana-VR) plant - 050ND08612. Follows the PROCEDURES for GOOD MANUFACTURING PRACTICE, according to the Code of Federal Regulations, Title 21, Volume 2, part 111, Certificate Number P2869.

Use of any information from the Technical Data Sheet and from CoA(s) is permitted with prior approval from Meetab.

Signed:

MEETAB SRL

Meetab srl Via Gabriele Rossetti 19, 20145 Milano P.IVA/CF 07790350966
Tel. +39 02 56566719 Fax +39 02 87162486,
mail: info@meetab.it, mail pec: meetab@pec.it sito: web site: www.meetab.it

FORMULA MY HEALTH

**Analysis for 1 tablet**	**mg**
Vitamin C	250
Vitamin E	60
Fruit Extract	50
Broccoli extract	50
L-Glutamine	50
Vitamin B3	35
of which Nicotinamide	30
of which Nicotinic Acid	5
Potassium	25
Vitamin B1	25
L-Lysine	25
trace Salts	20
L-Proline	20
Zinc	12,5
L-Glycine	10
N-Acetyl L-Cysteine	10
Vitamin B6	9
Manganese	8
Beta Carotene	7
Vitamin B5	5
Choline Bitartrate	5
Inositol	5
PABA	5
L-Arginine	5
L-Leucine	5
L-Histidine	5
L-isoleucine	5
L-Valine	5
L-Methionine	5
L-Tyrosine	5
Vitamin B2	4,8
L-Glutamic acid	2,5
L-Phenylalanine	2,5
L-Serine	2,5
L-Threonine	2,5
L-Alanine	2,5
L-Aspartic Acid	2,5
L-Citrulline	2,5
L-Taurine	2,5
L-Tryptophan	2,5

Meetab srl Via Gabriele Rossetti 19, 20145 Milano P.IVA/CF 07790350966
Tel. +39 02 56566719 Fax +39 02 87162486,
mail: info@meetab.it, mail pec: meetab@pec.it sito: web site: www.meetab.it

Copper	1,8
Methylfolate	0,4
Biotin	0,2
Chrome	0,2
Selenium	0,08
Sodium Molybdate	0,05
Vitamin K2 (Mena Q7)	0,045
Vitamin B12	0,03
Vitamin D3	0,025
Magnesium Citrate	10
Calcium	50

Meetab
your custom supplements

via G. Rossetti 19
20145 Milano (MI)
Italy

**CERTIFICATO D'ANALISI**<br>CERTIFICATE OF ANALYSIS	
**PRODOTTO**	**VITAMINA C ACIDO L-ASCORBICO USP/BP/FCC/E300**
**LOT.**	201701116
**DESCRIPTION**	WHITE ALMOST WHITE CRYSTALS – CRYSTALLYNE POWDER
**IDENTIFICATION**	POSITIVE (POSITIVE REACTION)
**MELTING POINT**	CONFORM (ABOUT 190°C)
**PH**	2.37
**SPECIFIC ROTATION**	21.04°
**CLARITY OF SOLUTION**	CLEAR
**COLOR OF SOLUTION**	< BY7
**COPPER**	<5 ppm
**HEAVY METALS**	<10 ppm
**MERCURY**	<0.1 mg/kg
**LEAD**	<2 mg/kg
**ARSENIC**	<3 ppm
**CADMIUM**	<1 mg/kg
**OXALIC ACID**	<0.2%
**IRON**	<2 ppm
**LOSS ON DRYING**	< 0.0%
**SULPHATE ASH**	<0.1%
**ORGANIC VOLATILE IMPURITIES**	PASS
**MESH**	PASS
**ASSAY**	99.75%
**TOTAL PLATE COUNT**	<10 cfu/g
**YEAST AND MOLDS**	<10 cfu/g
**E.COLI**	Absence/1 g
**SALMONELLA**	Absence/25 g
**COPY CONFORM TO THE ORIGINAL DOCUMENT – VALID ALTHOUGH NOT SIGNED**	

via G. Rossetti 19
20145 Milano (MI)
Italy

CERTIFICATO D'ANALISI<br>CERTIFICATE OF ANALYSIS	
**PRODOTTO**	**D-alpha tocopheryl acetate Vit E NAT**
**LOT.**	S20160701
**DESCRIPTION**	Almost white, yellowish or light brown, small particles
**IDENTIFICATION**	POSITIVE (POSITIVE REACTION)
**ASSAY**	703 IU
**LOSS ON DRYING**	2.46%
**BULK DENSITY**	0.480 g/cm3
**PARTICLE SIZE**	Residue on 40 meshes =/< 1% CONFORM
**BENZO(A)PYRENE**	<2 ppb
**HEAVY METALS (as Pb)**	<10 ppm
**MERCURY**	<0.1 mg/kg
**LEAD**	<2 mg/kg
**ARSENIC**	<1 mg/kg
**CADMIUM**	<1 mg/kg
**TOTAL BACTERIAL COUNT**	<1000 cfu/g CONFORM
**YEAST AND MOLDS**	<100 cfu/g CONFORM
**COLIFORM**	<0.3 MNP/g NEGATIVE
**SALMONELLA**	Negative/25 g
**STAPHYLOCOCCUS AUREUS**	Negative/1 g
**COPY CONFORM TO THE ORIGINAL DOCUMENT – VALID ALTHOUGH NOT SIGNED**	

via G. Rossetti 19
20145 Milano (MI)
Italy

**CERTIFICATO D'ANALISI**<br>CERTIFICATE OF ANALYSIS	
**PRODOTTO**	**NATURAL SOURCE VITAMIN E (MIXED NATURAL TOCOPHEROLS)**
**LOT.**	T(13590-0399)TPA
**APPEARANCE**	Clear, white to cream fine powder
**TASTE**	Bland, characteristic
**ODOUR**	Bland to none
**TOCHOFEROLS CONTENT**	min. 335 mg/g mixed tocopherols
**SPECIFIC GRAVITY/DENSITY (25°C)**	0. 58 g/cm3
**ACIDITY (as mixed tocopherols)**	<0.35 ml KOH 0.1N/f
**LOSS ON DRYING**	<5%
**BENZO(A)PYRENE**	<2 ppb
**HEAVY METALS (for mixed tocopherols)**	
**MERCURY**	<0.1 ppm
**LEAD**	<0.1 ppm
**ARSENIC**	<1 ppm
**CADMIUM**	< 1 ppm
**TOTAL AEROBIC COUNT**	<1000 cfu/g CONFORM
**YEAST AND MOLDS**	<100 cfu/g CONFORM
**E. COLI**	Negative cfu/g
**SALMONELLA**	Negative/25 g
**STAPHYLOCOCCUS AUREUS**	Negative/1 g
**COPY CONFORM TO THE ORIGINAL DOCUMENT – VALID ALTHOUGH NOT SIGNED**	

via G. Rossetti 19
20145 Milano (MI)
Italy

CERTIFICATO D'ANALISI<br>CERTIFICATE OF ANALYSIS	
**PRODUCT**	**VITAMIN PP -NICOTINAMIDE**
**LOT.**	OP11708013
**APPEARANCE**	Crystalline Powder
**COLOUR**	White
**IDENTIFICATION (IR,USP,EP,JP)**	Conforms
**CLARITY OF SOLUTION (5 g in 100 ml water)**	0.17
**pH (sol. 5 g in 100 ml water)**	6.4
**LOSS ON DRYING (EP)**	0.0% w/w
**MELTING RANGE (START/END)**	128.7-129.1°C
**PARTICLE SIZE FRACTION**	>50 micron 100% (=>90%)<br>> 250 micron 0% (<=8%)
**SULFATE ASH**	<0.05%
**CHLORIDE**	<70 mg/Kg
**SULFATE**	<190 mg/kg
**HEAVY METALS**	<20 mg/Kg
**ASSAY (HPLC)**	99.8%
**CONFORM TO CURRENT REQUIR OF EP,USP,FCC**	Conforms
**TOTAL PLATE COUNT**	Conforms
**YEAST & MOLD**	Conforms
**E. COLI**	Negative
**SALMONELLA**	Negative
**COPY CONFORM TO THE ORIGINAL DOCUMENT – VALID ALTHOUGH NOT SIGNED**	

Meetab
your custom supplements

via G. Rossetti 19
20145 Milano (MI)
Italy

CERTIFICATO D'ANALISI<br>CERTIFICATE OF ANALYSIS	
**PRODUCT**	**NICOTINIC ACID**
**LOT.**	16-17/NCN[P]/B/093
**APPEARANCE**	White cristalline powder
**SOLUBILITY**	Sparingly soluble in water, soluble in boiling water and in boiling ethanol (96%) and in dilute solution of alkali hydroxides and carbonates – Complies
**MELTING POINT**	237°C
**IR TEST**	Matches
**RELATED SUBSTANCE**	Complies
**HEAVY METAL**	<20 ppm
**LOSS ON DRYING**	0.23% w/w
**SULPHATED ASH/RESIDUE ON IGNITION**	0.027% w/w
**CHLORIDE**	<200ppm
**SULPHATE**	N.A.
**ASSAY**	99.75% w/w
**LEAD**	Less than 3mg/kg
**ARSENIC**	Less than 1mg/kg
**CADMIUM**	Less than 1mg/kg
**MERCURY**	Less than 0.1mg/kg
**TOTAL PLATE COUNT**	<30 cfu/g
**YEAST & MOLD**	< 10 cfu/g
**STAPHYLOCOCCUS AUREUS**	Negative
**E. COLI**	Negative
**SALMONELLA**	Negative
**COPY CONFORM TO THE ORIGINAL DOCUMENT – VALID ALTHOUGH NOT SIGNED**	

Meetab
your custom supplements

via G. Rossetti 19
20145 Milano (MI)
Italy

**CERTIFICATO D'ANALISI**<br>CERTIFICATE OF ANALYSIS	
**PRODUCT**	**VITAMIN B1 HCl (TIAMIN)**
**LOT.**	Y01201610005
**APPEARANCE**	White cristalline powder
**IDENTIFICATION**	Conforms
**ASPECT**	Conforms
**pH**	3.0
**SULPHATE**	<300 ppm
**NITRATE (ppm)**	Conforms
**HEAVY METAL**	<10 ppm
**LEAD**	<2 ppm
**ARSENIC**	< 3 ppm
**CADMIUM**	<1 ppm
**MERCURY**	<1 ppm
**ASSAY**	100%
**TOTAL PLATE COUNT**	<1000 cfu/g
**YEAST & MOLD**	< 100 cfu/g
**STAPHYLOCOCCUS AUREUS**	Negative
**E. COLI**	Negative
**SALMONELLA**	Negative
**CONCLUSION**	Product conform to EP-USP, FCC
**COPY CONFORM TO THE ORIGINAL DOCUMENT – VALID ALTHOUGH NOT SIGNED**	

Meetab
your custom supplements

via G. Rossetti 19
20145 Milano (MI)
Italy

**CERTIFICATO D'ANALISI**<br>CERTIFICATE OF ANALYSIS	
**PRODOTTO**	**VITAMIN K2 MK-7**
**LOT.**	1703
**ORIGIN**	BACILLUS LICHENIFORMIS; FERMENTED
**APPEARANCE**	POWDER
**COLOUR**	YELLOW TO PALE YELLOW
**ASSAY/RATIO:** VITAMIN K2 (MK-7)	Min. 2000 ppm (Observation: 2367 ppm)
**LOSS ON DRYING**	2.7%
**PARTICLE SIZE**	Min. 95% pass 40 mesh
**HEAVY METAL**	< 5 ppm
**ARSENIC**	<0.5 ppm
**TOTAL PLATE COUNT**	< 10,000 cfu/g
**YEAST AND MOULDS**	<100 cfu/g
**E.COLI**	Negative/10 g
**STAPHYLOCOCCUS AUREUS**	Negative/10 g
**SALMONELLA**	Negative/10 g
**COPY CONFORM TO THE ORIGINAL DOCUMENT – VALID ALTHOUGH NOT SIGNED**	

Meetab
your custom supplements

via G. Rossetti 19
20145 Milano (MI)
Italy

**CERTIFICATO D'ANALISI**<br>CERTIFICATE OF ANALYSIS	
**PRODUCT**	**PYRIDOXAL 5-PHOSPHATE**
**LOT.**	C041612804
**DESCRIPTION**	SLIGHTLY YELLOW OR OFF-WHITE POWDER
**IDENTIFICATION**	Conforms
**SOLUBILITY**	Conforms
**MELTING POINT**	142°c
**WATER by KF**	8.3%
**HEAVY METALS**	<10 ppm
**LEAD**	<1 ppm
**ARSENIC**	<1 ppm
**MERCURY**	<0.1 ppm
**CADMIUM**	<1 ppm
**pH (in 0.25% water)**	2.7
**PARTICLE SIZE (MESH)**	Conforms
**BULK DENSITY**	0.37 ml
**TAPPED DENSITY**	0.64 g/ml
**ASSAY (ON DRIED BASIS)**	99.2%
**RESIDUAL SOLVENT**	Conforms
**TOLENE**	Conforms
**TOTAL PLATE COUNT**	<100 cfu/g
**YEAST & MOLD**	<10 cfu/g
**E. COLI**	Negative
**SALMONELLA**	Negative
**COLIFORMS**	Negative
**COPY CONFORM TO THE ORIGINAL DOCUMENT – VALID ALTHOUGH NOT SIGNED**	

Meetab
your custom supplements

via G. Rossetti 19
20145 Milano (MI)
Italy

**CERTIFICATO D'ANALISI**<br>CERTIFICATE OF ANALYSIS	
**PRODOTTO**	**BETACAROTENE**
**LOT.**	6091711003
**APPEARANCE**	Cristalline Fine powder
**COLOR**	Brown/Red
**ODOUR**	Bland
**MELTING POINT**	177-179°C
**RESIDUAL SOLVENT**	Conforms
**IDENTIFICATION (BETACAROTENE)**	CONFORM
**IDENTIFICATION (IR)**	CONFORM
**LOSS ON DRYING**	0.01%
**HEAVY METALS**	<10 ppm CONFORM
**MERCURY**	<0.1 ppm CONFORM
**LEAD**	<2 ppm CONFORM
**ARSENIC**	<1 ppm CONFORM
**CADMIUM**	< 1 ppm CONFORM
**TOTAL AEROBIC COUNT**	<1000 cfu/g CONFORM (80 UFC/g)
**YEAST AND MOLDS**	<100 cfu/g CONFORM (<10 UFC/g)
**E. COLI**	Negative cfu/g
**SALMONELLA**	Negative/25 g
**TITTLE**	99.5% BETACAROTENE
**COPY CONFORM TO THE ORIGINAL DOCUMENT – VALID ALTHOUGH NOT SIGNED**	

Meetab
your custom supplements

via G. Rossetti 19
20145 Milano (MI)
Italy

CERTIFICATO D'ANALISI<br>CERTIFICATE OF ANALYSIS	
**PRODUCT**	**HYDROXOCOBALAMINE ACETATE – VIT. B12**
**LOT.**	A17243E
**APPEARANCE**	Crystalline powder or dark red crystals
**IDENTIFICATION**	Conforms
**A525/A531**	0.33
**A274/A351**	0.8
**pH**	6.0
**LOSS ON WEIGHT**	11.1%
**SUM OF IMPURITY**	<5.0%
**SPECTRUMPHOTOMETRIC ASSAY**	97.5% sps
**ACETONE (GC)**	738 ppm
**METHANOL (GC)**	<1000 ppm
**HEAVY METALS**	Conforms
**TOTAL PLATE COUNT**	<1000 cfu/g
**YEAST & MOLD**	<100 cfu/g
**E. COLI**	Negative
**SALMONELLA**	Negative
**BACTERIAN ENDOTOXINE**	<=0.4 Complies
**CONCLUSION**	This product meets with the specification of Ph. Eur
**COPY CONFORM TO THE ORIGINAL DOCUMENT – VALID ALTHOUGH NOT SIGNED**	

via G. Rossetti 19
20145 Milano (MI)
Italy

CERTIFICATO D'ANALISI<br>CERTIFICATE OF ANALYSIS	
**PRODUCT**	**VITAMIN B5 (D-CALCIUM PANTOTHENATE)**
**LOT.**	16061404
**APPEARANCE**	WHITE OR ALMOST WHITE POWDER
**INFRARED ABSORBATION**	Up to standard figure
**IDENTIFICATION OF CALCIUM ION**	Normal reaction
**pH VALUE**	Conform (6.8-8.0)
**ALKALINITY**	Conform
**SPECIFIC ROTATION**	+26.8°C
**LEAD**	<3 ppm
**ARSENIC**	<1 ppm
**CADMIUM**	<1 ppm
**MERCURY**	<0.1 ppm
**IMPURITY CONTENT**	Conform <1%
**HEAVY METALS**	<=0.002%
**NITROGEN CONTENT**	5.8%
**CALCIUM CONTENT**	8.3%
**LOSS ON DRYING**	2.2%
**RESIDUAL SOLVENT**	0.11%
**TOTAL PLATE COUNT**	<1000 cfu/g
**YEAST & MOLD**	<100 cfu/g
**E. COLI**	Negative/10 g
**SALMONELLA**	Negative/25 g
**ASSAY**	99.2%
**CONCLUSION**	Complies with USP39
**COPY CONFORM TO THE ORIGINAL DOCUMENT – VALID ALTHOUGH NOT SIGNED**	

via G. Rossetti 19
20145 Milano (MI)
Italy

**CERTIFICATO D'ANALISI**<br>CERTIFICATE OF ANALYSIS	
**PRODUCT**	**VITAMIN B2 (Riboflavin)**
**LOT.**	17D10-B11-PPR1717485
**APPEARANCE**	YELLOW- ORANGE POWDER
**IDENTIFICATION**	Conforms
**SPECIFIC ROTATION**	Conforms
**ABSORBANCE (Ph. Eur.)**	Conforms
**LUMIFLAVIN (USP)**	0.0052
**SULPHUR ASH**	0.08/100 g
**RESIDUE ON IGNITION**	0.08%
**LOSS ON DRYING**	1.0g/100g
**ASSAY**	99.9%
**HEAVY METALS**	<10 ppm
**LEAD**	<2 ppm
**ARSENIC**	<1 ppm
**CADMIUM**	<1 ppm
**MERCURY**	<0.1 ppm
**TOTAL PLATE COUNT**	<1000 cfu/g
**YEAST & MOLD**	<100 cfu/g
**E. COLI**	Negative/10 g
**SALMONELLA**	Negative/25 g
**PSUDOMONAS AERUGINOSA**	Negative/g
**STAPHYLOCOCCUS AUREUS**	Negative/g
**COPY CONFORM TO THE ORIGINAL DOCUMENT – VALID ALTHOUGH NOT SIGNED**	

Meetab
your custom supplements

via G. Rossetti 19
20145 Milano (MI)
Italy

**CERTIFICATO D'ANALISI**<br>CERTIFICATE OF ANALYSIS	
**PRODUCT**	**VITAMIN D3 100000 IU/g**
**LOT.**	5431-1712001
**APPEARANCE**	White to off White/Yellowish Powder
**IDENTIFICATION**	Conforms
**ASSAY**	Min. 100000 IU/g
**SIEVE ANALYSIS**	100% Pass 40 Mesh Conforms
**LOSS ON DRYING**	3.91%
**ARSENIC**	Conforms (<0.5 ppm)
**LEAD**	Conforms (0.5 ppm)
**CADMIUM**	Conforms (<0.1 ppm)
**MERCURY**	Conforms (<0.1 ppm)
**TOTAL PLATE COUNT**	10 cfu/g (<1000 cfu/g)
**YEAST & MOLD**	10 cfu/g (<100 cfu/g)
**E. COLI**	Negative
**SALMONELLA**	Negative
**STAPHYLOCOCCUS AUREUS**	Negative
**COLIFORMS**	Negative
**GMO/BSE/TSE/GLUTEN**	Absent
**COPY CONFORM TO THE ORIGINAL DOCUMENT – VALID ALTHOUGH NOT SIGNED**	

Meetab
your custom supplements

via G. Rossetti 19
20145 Milano (MI)
Italy

CERTIFICATO D'ANALISI<br>CERTIFICATE OF ANALYSIS	
**PRODUCT**	**D-BIOTIN (VIT H)**
**LOT.**	VH20160702C
**APPEARANCE**	White or off white crystalline powder
**SOLUBILITY**	Conforms
**IDENTIFICATION**	Conforms
**CLEAR AND COLOUR**	Conforms
**HEAVY METALS**	<10 ppm
**LEAD**	<2 ppm
**SULPHATED ASH**	0.07%
**SPECIFIC OPTICAL ROTATION**	90.7°
**MELTING POINT**	Conforms
**ORGANIC VOLATILE IMPURITY**	Conforms
**ASSAY**	99.9%
**RELATED SUBSTANCES**	Conforms
**LOSS ON DRYNG**	0.08%
**TOTAL PLATE COUNT**	<1000 cfu/g
**YEAST & MOLD**	<100 cfu/g
**E. COLI**	Negative
**SALMONELLA**	Negative
**CONCLUSION**	Confroms to USP38/EP8
**COPY CONFORM TO THE ORIGINAL DOCUMENT – VALID ALTHOUGH NOT SIGNED**	

via G. Rossetti 19
20145 Milano (MI)
Italy

CERTIFICATO D’ANALISI<br>CERTIFICATE OF ANALYSIS	
**PRODUCT**	**(6S)-5-METHYLTETRAHYDROFOLIC ACID, GLUCOSAMINE SALT (QUATREFOLIC®)**
**LOT.**	0001701113
**APPEARANCE**	Creamy to light brown powder
**MOLECULAR FORMULA**	C20H23N7O6(C6H14NO5)2
**MOLECULAR WEIGHT**	817.80
**IDENTIFICATION (IR)**	Conforms
**WATER CONTENT (K.F.)**	<=8.0%
**GLUCOSAMINE ASSAY ON D.B. (HPLC)**	34-36%
**5-METHYLTETRAHYDROFOLIC ACID ASSAY ON D.B. (HPLC)**	54-59%
**TOTAL IMPURITIES**	<=2.5%
**LEAD**	<=0.3 ppm
**ARSENIC**	<=1.5 ppm
**MERCURY**	<=0.1 ppm
**CADMIUM**	<=0.5 ppm
**BORON**	<=10 ppm
**TOTAL PLATE COUNT**	<100 cfu/g
**YEAST & MOLD**	<100 cfu/g
**E. COLI**	Negative/10 g
**SALMONELLA**	Negative 25/g
**ASSAY (ON DRIED BASIS)**	99.0%
**COPY CONFORM TO THE ORIGINAL DOCUMENT – VALID ALTHOUGH NOT SIGNED**	

Meetab
your custom supplements

via G. Rossetti 19
20145 Milano (MI)
Italy

**CERTIFICATO D'ANALISI**<br>CERTIFICATE OF ANALYSIS	
**PRODUCT**	**L-GLUTAMINE KYOWA QUALITY**
**LOT.**	GM-PL-17462
**APPEARANCE**	White Crystalline Powder
**IDENTIFICATION**	Conforms
**STATE OF SOLUTION**	NLT 99.2%
**pH**	5.1
**SPECIFIC ROTATION (AT 20°C)**	+7.0
**CHLORIDE**	NMT 0.020%
**SULFATE**	NMT 0.020%
**IRON**	NMT 100 ppm
**HAVY METALS**	NMT 5 ppm
**LEAD**	NMT 5 ppm
**ARSENIC**	NMT 1 ppm
**FOREIGN AMINO ACID**	NMT 0.5%
**LOSS ON DRYING**	0.01%
**RESIDUE OF IGNITION**	0.01%
**ASSAY (DRIED BASIS)**	99.5%
**TOTAL COUNT (CFU)**	NMT 1.000/g
**YEST AND MOLDS (CFU)**	NMT 100/g
**COLIFORM**	NEG
**INSOLUBLE FOREIGN MATTER**	Conforms
**COPY CONFORM TO THE ORIGINAL DOCUMENT – VALID ALTHOUGH NOT SIGNED**	

via G. Rossetti 19
20145 Milano (MI)
Italy

CERTIFICATO D'ANALISI<br>CERTIFICATE OF ANALYSIS	
**PRODOTTO**	**Amino acid Mix:** L-Lysine HCl, L-Proline, L-Glycine, N-acetyl-L-Cysteine, L-Arginine, L-Leucine, L-Histidine, L-Isoleucine, L- Valine, L-Methionine, L-Tyrosine, L-Glutamic Acid, L-Phenylalanine, L-Serine, L-Threonine, L-Alanine, L-Aspartic Acid, L-Citrulline, L-Taurine, L-Tryptophan
**LOT.**	201217
**APPEARANCE**	White crystalline powder or colorless crystals
**STATE OF SOLUTION**	>95%
**CHLORIDE**	<=0.02%
**AMMONIUM**	<=0.02%
**SULFATE**	<=0.02%
**MERCURY**	<0.1 ppm
**LEAD**	< 5 ppm
**ARSENIC**	<1 ppm
**CADMIUM**	< 1 ppm
**HEAVY METALS**	<= 10 ppm
**LOSS ON DRYING**	<=0.2%
**RESIDUE ON IGNITION**	<=0.10%
**TOTAL PLATE COUNT**	<1000 cfu/g CONFORM
**YEAST AND MOLDS**	<100 cfu/g CONFORM
**E. COLI**	Negative cfu/g
**SALMONELLA**	Negative/25 g
**ASSAY**	99.5%
**CONCLUSION**	Product conform to USP36/USP/24FCC/AJI92/JP15
**COPY CONFORM TO THE ORIGINAL DOCUMENT(s) – VALID ALTHOUGH NOT SIGNED**	

Meetab
your custom supplements

via G. Rossetti 19
20145 Milano (MI)
Italy

**CERTIFICATO D'ANALISI**<br>CERTIFICATE OF ANALYSIS	
**PRODUCT**	**TRI-MAGNESIUM CITRATE-NONHYDRATE**
**LOT.**	4210065
**APPEARANCE**	White crystalline powder
**IDENTIFICATION**	Pass test
**pH (5%)**	6.8
**CALCIUM**	Less than 1%
**CHLORIDE**	Less than 500 ppm
**IRON**	Less than 200 ppm
**LOD**	27.48%
**LEAD**	Less than 0.5 ppm
**OXALATE**	Less than 100 ppm
**SULFATE**	Less than 0.2%
**HEAVY METALS**	Less than 5 ppm
**Mg (on dry basis)**	16%
**COPY CONFORM TO THE ORIGINAL DOCUMENT – VALID ALTHOUGH NOT SIGNED**	

via G. Rossetti 19
20145 Milano (MI)
Italy

**CERTIFICATO D'ANALISI**<br>CERTIFICATE OF ANALYSIS	
**PRODOTTO**	**POTASSIO CITRATO**
**LOT.**	17I05-B16
**LOSS ON DRYING**	<6% (5.6%)
**APPEARANCE**	White crystalline powder or colorless crystals
**IDENTIFICATION A**	CONFORM
**TITTLE**	99.4%
**Ph**	8.6
**OSSALATI**	CONFORM
**MERCURY**	<1 ppm CONFORM
**LEAD**	<2 ppm CONFORM
**ARSENIC**	<1 ppm CONFORM
**HEAVY METALS**	<5 ppm CONFORM
**TOTAL AEROBIC COUNT**	CONFORM
**YEAST AND MOLDS**	CONFORM
**COPY CONFORM TO THE ORIGINAL DOCUMENT – VALID ALTHOUGH NOT SIGNED**	

via G. Rossetti 19
20145 Milano (MI)
Italy

**CERTIFICATO D'ANALISI**<br>CERTIFICATE OF ANALYSIS	
**PRODOTTO**	**MANGANESE GLUCONATO**
**LOT.**	20141101
**APPEARANCE**	Fine powder
**COLOR**	ALMOST WHITE/ROSE
**IDENTIFICATION**	CONFORM
**UMIDITA'**	6.8%
**SPLFATI**	0.02%
**CLORURI**	conform
**LEAD**	<5 ppm CONFORM
**CADMIUM**	< 3 ppm CONFORM
**SOST. RIDUCENTI**	0.2%
**IMPUREZZE ORGANICHE VOLATILI**	CONFORME
**HEAVY METALS**	<20 ppm CONFORM
**TITOLO SU BASE ANIDRA**	99.8%
**CONTENUTO DI MANGANESE**	12.31%
**TOTAL AEROBIC COUNT**	<1000 cfu/g CONFORM
**YEAST AND MOLDS**	<100 cfu/g CONFORM
**E. COLI**	Negative cfu/g
**SALMONELLA**	Negative/25 g
**GLUTEN**	FREE
**COPY CONFORM TO THE ORIGINAL DOCUMENT – VALID ALTHOUGH NOT SIGNED**	

Meetab
your custom supplements

via G. Rossetti 19
20145 Milano (MI)
Italy

**CERTIFICATO D'ANALISI**<br>CERTIFICATE OF ANALYSIS	
**PRODOTTO**	**OXXYNEA® (MIX SOLUBILE DI ESTRATTI DA FRUTTA E VERDURA)**
**LOT.**	OXFP170316
**TIPO DI PREPARAZIONE UTILIZZATA**	Polvere ad alto potere antiossidante ottenuta da una miscela di frutti e verdure standardizzata in valore ORAC (1 g = 3500 valori ORAC)
**ORIGINE**	France, Spain, Italy
**ASPECT**	Fine powder
**COLOUR**	Brown
**TASTE**	Characteristic
**ODOR**	Typical
**COMPOSITION**	Estratto da frutta 40-50%: Semi d'uva (rossa, bianca), Arancia, Pompelmo, Papaia, Ananas, Fragole, Ciliegie, Mela, Albicocca, Mirtillo, Ribes nero.<br>Estratto da vegetali: 30-40%: Pomodoro, Carote, Cocomero, Tè verde, Broccoli, Cavolo, Cipolla, Aglio, Asparago, Olive, Cetriolo.<br>Maltodestrina 10-20%
**TOTAL POLYPHENOLS (% CATECHINE EQ.)**	83.41%
**ORAC VALUE (µmol TE/g)**	11773.1%
**LOSS ON DRYING %**	0.1%
**ASH %**	1.5%
**SOLUBILITY (1% W/V)**	CONFORMS
**Ph (10% W/V)**	4.4
**BULK DENSITY**	0.50
**TAPPED DENSITY**	0.70
**MESH SIZE (US mesh)**	CONFORMS
**ARSENIC (As)**	<0.1 ppm
**CADMIUM (Cd)**	0.01% ppm
**MERCURY (Hg)**	<0.005 ppm
**LEAD (Pb)**	0.06 ppm
**TOTAL PLATE COUNT**	<1000 cfu/g CONFORM
**YEAST AND MOLDS**	<100 cfu/g CONFORM
**COLIFORMS**	Negative cfu/1 g
**SALMONELLA**	Negative/25 g
**STAPHYLOCOCCUS AUREUS**	Negative cfu/1 g
**COPY CONFORM TO THE ORIGINAL DOCUMENT – VALID ALTHOUGH NOT SIGNED**	

Meetab
your custom supplements

via G. Rossetti 19
20145 Milano (MI)
Italy

**CERTIFICATO D'ANALISI**<br>CERTIFICATE OF ANALYSIS	
**PRODUCT NAME**	**BROCCOLI 1:25**
**LOT.**	NUT/16/0896
**BOTANICAL NAME**	Brassica oleracea L.
**EXTRACTION SOLVENT**	Water
**E/D RATIO**	1:25
**EXCIPIENT**	Maltodextrin from maize
**AUXILIARY SUBSTANCE**	<=0.5% colloidal anydrous silica
**PREPARATION TYPE**	Dry extract
**PART OF PLANT USED**	Leaves
**ORIGIN**	Italy
**ASPECT**	Powder
**COLOUR**	Brownish to greenish
**TASTE**	Characteristic
**ODOR**	Characteristic
**IDENTIFICATION**	Complies (met. TLC)
**PARTICLE SIZE**	>=90% through 35 Mesh (500 micron)
**DENSITY**	0.5 g/ml ~
**LOSS ON DRYING**	<=5%
**HEAVY METALS**	<10 ppm<br>Pb <3 ppm; Cd <1 ppm; Hg <0.1 ppm
**RESIDUAL SOLVENTS**	Cpmplies to Directive 2009/32/EC
**PESTICIDES**	Complies to Reg. 2008/839/CE
**POLYCYCLIC AROMATIC HYDROCARBONS**	Conforms to Reg. UE 1933/2015
**AFLATOXINS**	Aflatoxin B1: <5 ppb<br>Total aflatoxin (B1,B2,G1,G2): <10 ppb
**BACTERIAL COUNT (TAMC)**	<= 5x10000 cfu/g
**YEAST AND MOUDS (TYMC)**	<= 5x10 cfu/g
**PATHOGENS**	Salmonella: absent/25g<br>E. Coli: absent/1 g
**ENTEROBACTERIACEE**	<=100 cfu/g
**GLUTEN FREE**	Yes
**COPY CONFORM TO THE ORIGINAL DOCUMENT – VALID ALTHOUGH NOT SIGNED**	

via G. Rossetti 19
20145 Milano (MI)
Italy

**CERTIFICATO D'ANALISI**<br>CERTIFICATE OF ANALYSIS	
**PRODUCT**	**DICALCIUM PHOSPHATE 2 HYDRATE**
**LOT.**	C51769A
**APPEARANCE**	White Crystalline Powder
**IDENTIFICATION**	Conforms
**ASSAY**	99.5%
**pH**	8.0
**P205**	43%
**LOSS ON IGNITION (800°C)**	26.1%
**ALUMINIUM**	Conforms (<200 ppm)
**ARSENIC**	Conforms (<1 ppm)
**LEAD**	Conforms (0.15 ppm)
**CADMIUM**	Conforms (<1 ppm)
**IRON**	Conforms (<400 ppm)
**MERCURY**	Conforms (<1 ppm)
**HEAVY METALS (as Pb)**	Conforms (<30 ppm)
**BARIUM-TEST**	Conforms
**CHLORIDE**	Conforms (<0.25%)
**FLUORIDE**	Conforms (<50 ppm)
**SULFATE**	Conforms (<0.16%)
**CARBONETE-TEST**	Conforms
**HCl-INSOLUBLE SUBSTANCE**	<0.05%
**FILTER TEST**	Conforms
**CONCLUSION**	Product conform to USP/FCC/EP
**GMO/BSE/TSE/GLUTEN**	Absent
**COPY CONFORM TO THE ORIGINAL DOCUMENT – VALID ALTHOUGH NOT SIGNED**	

via G. Rossetti 19
20145 Milano (MI)
Italy

**CERTIFICATO D'ANALISI**<br>CERTIFICATE OF ANALYSIS	
**PRODUCT**	**SEA MINERAL BLEND (CONTAINING TRACE MINERALS AND ELEMENTS)**
**LOT.**	004-G02
**CHLORIDE**	381 mg/g
**MAGNESIUM**	128 mg/g
**SULFATE**	56.5 mg/g
**SODIUM**	15.9 mg/g
**POTASSIUM**	22.7 mg/g
**LITHIUM**	0.32 mg/g
**BORON**	0.090 mg/g
**CALCIUM**	0.060 mg/g
**This product also contain other naturally occurring trace Minerals found in the Great Salt Lake**	
**PERCENT AMBIENT MOISTURE**	0.30%
**PERCENT TOTAL MOISTURE**	7.3%
**80 MESH SIZE**	90.8% Weight^
**STANDARD PLATE COUNT**	<1000 cfu/g
**TOTAL COLIFORM**	<100 cfu/g
**ESCHERICHIA COLI**	Negative
**ENTEROBACTERIACEAE**	Negative
**STAPHYLOCOCCUS AUREUS**	Negative
**PSUDOMONAS AERUGINOSA**	Negative
**YEAST & FUNGUS**	<100 cfu/g
**COPY CONFORM TO THE ORIGINAL DOCUMENT – VALID ALTHOUGH NOT SIGNED**	

via G. Rossetti 19
20145 Milano (MI)
Italy

CERTIFICATO D'ANALISI<br>CERTIFICATE OF ANALYSIS	
**PRODOTTO**	**CALCIO CARBONATO PESANTE**
**LOT.**	16J14-B04
**IDENTIFICAZIONE A+B**	POSITIVE (POSITIVE REACTION)
**CONTENUTO CaCO3**	99.6%
**INSOLUBILE IN ACIDO**	<0.2%
**CLORURI**	<330 ppm
**SO4**	<0.25%
**As**	<3 ppm
**Ba**	CONFORM
**HEAVY METAL**	<20 ppm
**Fe**	<200 ppm
**MG e METALLI ALCALINO-TERROSI**	<1%
**LOSS ON DRYING**	<2%
**Pb**	<3 ppm
**Cd**	<1 ppm
**Sb, Cu, Cr, Zn, Ba (E170)**	<100 ppm
**F**	<50 ppm
**Hg**	<0.1 ppm
**OVI**	CONFORM
**COPY CONFORM TO THE ORIGINAL DOCUMENT – VALID ALTHOUGH NOT SIGNED**	

via G. Rossetti 19
20145 Milano (MI)
Italy

**CERTIFICATO D'ANALISI**<br>CERTIFICATE OF ANALYSIS	
**PRODUCT**	**L-SELENIOMETHIONINE**
**LOT.**	2017423
**APPEARANCE**	Fine powder
**COLOUR**	White
**ODOR**	Characteristic
**IDENTIFICATION**	Conform (IR)
**MOISTURE**	Conform
**LEAD**	<0.1 ppm
**CADMIUM**	<0.1 ppm
**MERCURY**	<0.1 ppm
**ARSENIC**	<0.1 ppm
**THROUGH SCREEN 60 MESH**	Conform
**TOTAL PLATE COUNT**	<1000 cfu/g
**YEAST & MOLD**	< 100 cfu/g
**STAPHYLOCOCCUS AUREUS**	Negative/g
**E. COLI**	Negative/10 g
**SALMONELLA**	Negative/25 g
**PSUDOMONAS AERUGINOSA**	Negative/g
**SELENIUM CONTENT**	=>5000 ppm
**COPY CONFORM TO THE ORIGINAL DOCUMENT – VALID ALTHOUGH NOT SIGNED**	

Meetab
your custom supplements

via G. Rossetti 19
20145 Milano (MI)
Italy

CERTIFICATO D'ANALISI<br>CERTIFICATE OF ANALYSIS	
**PRODUCT**	**COPPER GLUCONATE**
**LOT.**	17C10-B12-PPR1711081
**IDENTIFICATION**	Conform
**MOISTURE**	Conform
**ASSAY (ON DRY BASIS)**	98.5%
**CHLORIDE**	0.03%
**SULPHATE**	0.03%
**ARSENIC**	1.3 ppm
**LEAD**	<5 ppm
**REDUCING SUBSTANCES**	0.7%
**COPPER CONTENT**	13.79%
**80 MESH SIZE**	>090%
**TOTAL PLATE COUNT**	20 cfu/g
**YEAST & MOLD**	10 cfu/g
**E. COLI**	Negative/10 g
**SALMONELLA**	Negative/25 g
**COPY CONFORM TO THE ORIGINAL DOCUMENT – VALID ALTHOUGH NOT SIGNED**	

Meetab
your custom supplements

via G. Rossetti 19
20145 Milano (MI)
Italy

**CERTIFICATO D'ANALISI**<br>CERTIFICATE OF ANALYSIS	
**PRODUCT**	**BITARTRATE CHOLINE**
**LOT.**	3012864
**APPEARANCE**	WHITE CRYSTALLINE POWDER
**IDENTIFICATION**	COMPLIES (IR, B)
**SPECIFIC ROTATION**	+17.9°
**pH (10%)**	3.4
**WATER**	0.07%
**RESIDUE ON IGNITION**	<0.1%
**AS**	<2 ppm
**LEAD**	<0.3 ppm
**TOTAL AMINES**	4.1 ppm
**CROMATOGRAPHIC PURITY**	Conforms
**O.V.I.**	Conforms
**1.4 DIOXANE**	Conforms
**HEAVY METAL**	<10 ppm
**ASSAY (anhydrous basis)**	99.52%
**TOTAL AEROBIC COUNT**	<1000 cfu/g
**YEAST & MOLD**	<100 cfu/g
**E. COLI**	Negative/10 g
**SALMONELLA**	Negative/25 g
**CONFORM**	USP/DAB
**COPY CONFORM TO THE ORIGINAL DOCUMENT – VALID ALTHOUGH NOT SIGNED**	

via G. Rossetti 19
20145 Milano (MI)
Italy

CERTIFICATO D'ANALISI<br>CERTIFICATE OF ANALYSIS	
**PRODUCT**	**INOSITOL**
**LOT.**	HZ-IN1702065
**APPEARANCE**	WHITE CRYSTALLINE POWDER
**IDENTIFICATION**	Positive reaction (A,B)
**MELTING POINT**	225.3-226.4°C
**LOSS ON DRYING**	0.02%
**RESIDUE ON IGNITION**	0.02%
**CLARITY/COLOR OF SOLUTION**	meet the requirement
**CHLORIDE**	<0.005%
**SULFATE**	<0.006%
**CALCIUM**	meet the requirement
**HEAVY METALS**	<5 ppm
**LEAD**	<0.5 ppm
**ARSENIC**	<0.5 ppm
**CADMIUM**	<0.5 ppm
**MERCURY**	<0.1 ppm
**TOTAL PLATE COUNT**	<10 cfu/g
**YEAST & MOLD**	<10 cfu/g
**E. COLI**	Negative/10 g
**SALMONELLA**	Negative/25 g
**ASSAY**	98.59%
**CONCLUSION**	The good are complied with FCCVIX/USP38/NF33
**COPY CONFORM TO THE ORIGINAL DOCUMENT – VALID ALTHOUGH NOT SIGNED**	

via G. Rossetti 19
20145 Milano (MI)
Italy

CERTIFICATO D'ANALISI<br>CERTIFICATE OF ANALYSIS	
**PRODUCT**	**PARA-AMINOBENZOIC ACID**
**LOT.**	17E15-B09
**APPEARANCE**	WHITE CRYSTALLINE POWDER
**IDENTIFICATION**	Conforms
**PARTICLE SIZE (MESH)**	Conforms
**ASSAY (ON DRIED BASIS)**	99.75%
**RESIDUE ON IGNITION**	0.01%
**LOSS ON DRYING**	0.08%
**HUMIDITY**	1.1%
**HEAVY METALS**	<20 ppm
**TOTAL PLATE COUNT**	<1000 cfu/g
**MERCURY**	<0.1 ppm
**CADMIUM**	<1 ppm
**YEAST & MOLD**	<100 cfu/g
**E. COLI**	Negative/1g
**SALMONELLA**	Negative/25g
**CHEMICAL FORMULA**	C7H7N02
**COPY CONFORM TO THE ORIGINAL DOCUMENT – VALID ALTHOUGH NOT SIGNED**	

via G. Rossetti 19
20145 Milano (MI)
Italy

CERTIFICATO D'ANALISI<br>CERTIFICATE OF ANALYSIS	
**PRODUCT**	**SODIUM MOLYBDATE 10% ON MALTODEXTRINE**
**LOT.**	0000811963
**MOLYBDENUM (%)**	4%
**LOSS ON DRYING (2h – 105°C)**	4.2%
**APPEARANCE**	WHITE CRYSTALLINE POWDER
**HEAVY METALS**	Conforms
**TOTAL AEROBIC COUNT 30°C max 1000/g**	Conforms
**MOULD max 100/g**	Conforms
**YEAST max 100/g**	Conforms
**COLIFORM GERMS/ENTEROBACTERIACEAE/10g**	Negative
**E. COLI/10 g**	Negative
**SALMONELLA SPECIES/100 g**	Negative
**COPY CONFORM TO THE ORIGINAL DOCUMENT – VALID ALTHOUGH NOT SIGNED**	

via G. Rossetti 19
20145 Milano (MI)
Italy

**CERTIFICATO D'ANALISI**<br>CERTIFICATE OF ANALYSIS	
**PRODUCT**	**CHROMIUM PICOLINATE**
**LOT.**	17J05-B26-PPR1804210
**APPEARANCE**	RED-VIOLET CRYSTALLINE POWDER
**IDENTIFICATION**	Conforms
**PARTICLE SIZE (MESH)**	Conforms
**pH**	6.2
**BULK DENSITY**	0.65 g/ml
**ASSAY (ON DRIED BASIS)**	99.2%
**CHLORIDE**	0.002%
**SULFATE**	0.03%
**HUMIDITY**	1.1%
**HEAVY METALS**	<10 ppm
**LEAD**	<3 ppm
**ARSENIC**	<5 ppm
**MERCURY**	<0.1 ppm
**CADMIUM**	<1 ppm
**TOTAL PLATE COUNT**	<1000 cfu/g
**YEAST & MOLD**	<100 cfu/g
**E. COLI**	Negative/1g
**SALMONELLA**	Negative/1g
**CHEMICAL FORMULA**	$Cr(C_6H_4NO_2)_3$
**CHROMIUM CONTENT**	12.29%
**COPY CONFORM TO THE ORIGINAL DOCUMENT – VALID ALTHOUGH NOT SIGNED**	

FORMULA MY ANTIOXIDANT

**Analysis for 4 tablets**	**mg**
Vitamin C	640
Green Tea dry extract	500
L-Lysine	500
Alpha Lipoic Acid	150
Quercetin	150
L-Glutamine	100
L-Methionine	100
L-Proline	100
L-Arginine	100
N-Acetil L-Cisteina	100
Broccoli extract	100
Fruit Extract	100
Vitamin E	60
Prunus cerasus e.s.	50
Bioflavonoids from citrus	50
Resveratrol	50
L-Glycine	50
Vitamin B3	35
(of which Nicotinamide)	30
(of which Nicotinic Acid)	5
Vitamin B1	25
Acerola	20
Bromelain	10
Lutein	10
Picogenolo	10
Vitamin B6	9
Beta Carotene	7
Vitamin B5	5
Choline Bitartrate	5
Inositol	5
PABA	5
Vitamin B2	4,8
Astaxanthin	4
Vitamin B12	0,033
Biotin	0,2
Methylfolate (Folic Acid)	0,1
Vitamin D3	0,025

Meetab srl Via Gabriele Rossetti 19, 20145 Milano P.IVA/CF 07790350966
Tel. +39 02 56566719 Fax +39 02 87162486,
mail: info@meetab.it, mail pec: meetab@pec.it sito: web site: www.meetab.it

via G. Rossetti 19
20145 Milano (MI)
Italy

CERTIFICATO D'ANALISI<br>CERTIFICATE OF ANALYSIS	
**PRODOTTO**	**VITAMINA C ACIDO L-ASCORBICO USP/BP/FCC/E300**
**LOT.**	201701116
**DESCRIPTION**	WHITE ALMOST WHITE CRYSTALS – CRYSTALLYNE POWDER
**IDENTIFICATION**	POSITIVE (POSITIVE REACTION)
**MELTING POINT**	CONFORM (ABOUT 190°C)
**PH**	2.37
**SPECIFIC ROTATION**	21.04°
**CLARITY OF SOLUTION**	CLEAR
**COLOR OF SOLUTION**	< BY7
**COPPER**	<5 ppm
**HEAVY METALS**	<10 ppm
**MERCURY**	<0.1 mg/kg
**LEAD**	<2 mg/kg
**ARSENIC**	<3 ppm
**CADMIUM**	<1 mg/kg
**OXALIC ACID**	<0.2%
**IRON**	<2 ppm
**LOSS ON DRYING**	< 0.0%
**SULPHATE ASH**	<0.1%
**ORGANIC VOLATILE IMPURITIES**	PASS
**MESH**	PASS
**ASSAY**	99.75%
**TOTAL PLATE COUNT**	<10 cfu/g
**YEAST AND MOLDS**	<10 cfu/g
**E.COLI**	Absence/1 g
**SALMONELLA**	Absence/25 g
**COPY CONFORM TO THE ORIGINAL DOCUMENT – VALID ALTHOUGH NOT SIGNED**	

via G. Rossetti 19
20145 Milano (MI)
Italy

CERTIFICATO D'ANALISI<br>CERTIFICATE OF ANALYSIS	
**PRODOTTO**	**D-alpha tocopheryl acetate Vit E NAT**
**LOT.**	S20160701
**DESCRIPTION**	Almost white, yellowish or light brown, small particles
**IDENTIFICATION**	POSITIVE (POSITIVE REACTION)
**ASSAY**	703 IU
**LOSS ON DRYING**	2.46%
**BULK DENSITY**	0.480 g/cm3
**PARTICLE SIZE**	Residue on 40 meshes =/< 1% CONFORM
**BENZO(A)PYRENE**	<2 ppb
**HEAVY METALS (as Pb)**	<10 ppm
**MERCURY**	<0.1 mg/kg
**LEAD**	<2 mg/kg
**ARSENIC**	<1 mg/kg
**CADMIUM**	<1 mg/kg
**TOTAL BACTERIAL COUNT**	<1000 cfu/g CONFORM
**YEAST AND MOLDS**	<100 cfu/g CONFORM
**COLIFORM**	<0.3 MNP/g NEGATIVE
**SALMONELLA**	Negative/25 g
**STAPHYLOCOCCUS AUREUS**	Negative/1 g
**COPY CONFORM TO THE ORIGINAL DOCUMENT – VALID ALTHOUGH NOT SIGNED**	

via G. Rossetti 19
20145 Milano (MI)
Italy

CERTIFICATO D'ANALISI<br>CERTIFICATE OF ANALYSIS	
**PRODOTTO**	**NATURAL SOURCE VITAMIN E (MIXED NATURAL TOCOPHEROLS)**
**LOT.**	T(13590-0399)TPA
**APPEARANCE**	Clear, white to cream fine powder
**TASTE**	Bland, characteristic
**ODOUR**	Bland to none
**TOCHOFEROLS CONTENT**	min. 335 mg/g mixed tocopherols
**SPECIFIC GRAVITY/DENSITY (25°C)**	0. 58 g/cm3
**ACIDITY (as mixed tocopherols)**	<0.35 ml KOH 0.1N/f
**LOSS ON DRYING**	<5%
**BENZO(A)PYRENE**	<2 ppb
**HEAVY METALS (for mixed tocopherols)**	
**MERCURY**	<0.1 ppm
**LEAD**	<0.1 ppm
**ARSENIC**	<1 ppm
**CADMIUM**	< 1 ppm
**TOTAL AEROBIC COUNT**	<1000 cfu/g CONFORM
**YEAST AND MOLDS**	<100 cfu/g CONFORM
**E. COLI**	Negative cfu/g
**SALMONELLA**	Negative/25 g
**STAPHYLOCOCCUS AUREUS**	Negative/1 g
**COPY CONFORM TO THE ORIGINAL DOCUMENT – VALID ALTHOUGH NOT SIGNED**	

Meetab
your custom supplements

via G. Rossetti 19
20145 Milano (MI)
Italy

**<u>CERTIFICATO D'ANALISI</u>**<br>CERTIFICATE OF ANALYSIS	
**PRODUCT**	**VITAMIN PP -NICOTINAMIDE**
**LOT.**	OP11708013
**APPEARANCE**	Crystalline Powder
**COLOUR**	White
**IDENTIFICATION (IR,USP,EP,JP)**	Conforms
**CLARITY OF SOLUTION (5 g in 100 ml water)**	0.17
**pH (sol. 5 g in 100 ml water)**	6.4
**LOSS ON DRYING (EP)**	0.0% w/w
**MELTING RANGE (START/END)**	128.7-129.1°C
**PARTICLE SIZE FRACTION**	>50 micron 100% (=>90%)<br>> 250 micron 0% (<=8%)
**SULFATE ASH**	<0.05%
**CHLORIDE**	<70 mg/Kg
**SULFATE**	<190 mg/kg
**HEAVY METALS**	<20 mg/Kg
**ASSAY (HPLC)**	99.8%
**CONFORM TO CURRENT REQUIR OF EP,USP,FCC**	Conforms
**TOTAL PLATE COUNT**	Conforms
**YEAST & MOLD**	Conforms
**E. COLI**	Negative
**SALMONELLA**	Negative
**COPY CONFORM TO THE ORIGINAL DOCUMENT – VALID ALTHOUGH NOT SIGNED**	

via G. Rossetti 19
20145 Milano (MI)
Italy

CERTIFICATO D'ANALISI<br>CERTIFICATE OF ANALYSIS	
**PRODUCT**	**NICOTINIC ACID**
**LOT.**	16-17/NCN[P]/B/093
**APPEARANCE**	White cristalline powder
**SOLUBILITY**	Sparingly soluble in water, soluble in boiling water and in boiling ethanol (96%) and in dilute solution of alkali hydroxides and carbonates – Complies
**MELTING POINT**	237°C
**IR TEST**	Matches
**RELATED SUBSTANCE**	Complies
**HEAVY METAL**	<20 ppm
**LOSS ON DRYING**	0.23% w/w
**SULPHATED ASH/RESIDUE ON IGNITION**	0.027% w/w
**CHLORIDE**	<200ppm
**SULPHATE**	N.A.
**ASSAY**	99.75% w/w
**LEAD**	Less than 3mg/kg
**ARSENIC**	Less than 1mg/kg
**CADMIUM**	Less than 1mg/kg
**MERCURY**	Less than 0.1mg/kg
**TOTAL PLATE COUNT**	<30 cfu/g
**YEAST & MOLD**	< 10 cfu/g
**STAPHYLOCOCCUS AUREUS**	Negative
**E. COLI**	Negative
**SALMONELLA**	Negative
**COPY CONFORM TO THE ORIGINAL DOCUMENT – VALID ALTHOUGH NOT SIGNED**	

Meetab
your custom supplements

via G. Rossetti 19
20145 Milano (MI)
Italy

CERTIFICATO D'ANALISI<br>CERTIFICATE OF ANALYSIS	
**PRODUCT**	**VITAMIN B1 HCl (TIAMIN)**
**LOT.**	Y01201610005
**APPEARANCE**	White cristalline powder
**IDENTIFICATION**	Conforms
**ASPECT**	Conforms
**pH**	3.0
**SULPHATE**	<300 ppm
**NITRATE (ppm)**	Conforms
**HEAVY METAL**	<10 ppm
**LEAD**	<2 ppm
**ARSENIC**	< 3 ppm
**CADMIUM**	<1 ppm
**MERCURY**	<1 ppm
**ASSAY**	100%
**TOTAL PLATE COUNT**	<1000 cfu/g
**YEAST & MOLD**	< 100 cfu/g
**STAPHYLOCOCCUS AUREUS**	Negative
**E. COLI**	Negative
**SALMONELLA**	Negative
**CONCLUSION**	Product conform to EP-USP, FCC
**COPY CONFORM TO THE ORIGINAL DOCUMENT – VALID ALTHOUGH NOT SIGNED**	

via G. Rossetti 19
20145 Milano (MI)
Italy

**CERTIFICATO D'ANALISI**<br>CERTIFICATE OF ANALYSIS	
**PRODUCT**	**PYRIDOXAL 5-PHOSPHATE**
**LOT.**	C041612804
**DESCRIPTION**	SLIGHTLY YELLOW OR OFF-WHITE POWDER
**IDENTIFICATION**	Conforms
**SOLUBILITY**	Conforms
**MELTING POINT**	142°c
**WATER by KF**	8.3%
**HEAVY METALS**	<10 ppm
**LEAD**	<1 ppm
**ARSENIC**	<1 ppm
**MERCURY**	<0.1 ppm
**CADMIUM**	<1 ppm
**pH (in 0.25% water)**	2.7
**PARTICLE SIZE (MESH)**	Conforms
**BULK DENSITY**	0.37 ml
**TAPPED DENSITY**	0.64 g/ml
**ASSAY (ON DRIED BASIS)**	99.2%
**RESIDUAL SOLVENT**	Conforms
**TOLENE**	Conforms
**TOTAL PLATE COUNT**	<100 cfu/g
**YEAST & MOLD**	<10 cfu/g
**E. COLI**	Negative
**SALMONELLA**	Negative
**COLIFORMS**	Negative
**COPY CONFORM TO THE ORIGINAL DOCUMENT – VALID ALTHOUGH NOT SIGNED**	

Meetab
your custom supplements

via G. Rossetti 19
20145 Milano (MI)
Italy

**CERTIFICATO D'ANALISI**<br>CERTIFICATE OF ANALYSIS	
**PRODOTTO**	**BETACAROTENE**
**LOT.**	6091711003
**APPEARANCE**	Cristalline Fine powder
**COLOR**	Brown/Red
**ODOUR**	Bland
**MELTING POINT**	177-179°C
**RESIDUAL SOLVENT**	Conforms
**IDENTIFICATION (BETACAROTENE)**	CONFORM
**IDENTIFICATION (IR)**	CONFORM
**LOSS ON DRYING**	0.01%
**HEAVY METALS**	<10 ppm CONFORM
**MERCURY**	<0.1 ppm CONFORM
**LEAD**	<2 ppm CONFORM
**ARSENIC**	<1 ppm CONFORM
**CADMIUM**	< 1 ppm CONFORM
**TOTAL AEROBIC COUNT**	<1000 cfu/g CONFORM (80 UFC/g)
**YEAST AND MOLDS**	<100 cfu/g CONFORM (<10 UFC/g)
**E. COLI**	Negative cfu/g
**SALMONELLA**	Negative/25 g
**TITTLE**	99.5% BETACAROTENE
**COPY CONFORM TO THE ORIGINAL DOCUMENT – VALID ALTHOUGH NOT SIGNED**	

via G. Rossetti 19
20145 Milano (MI)
Italy

**CERTIFICATO D'ANALISI** CERTIFICATE OF ANALYSIS	
**PRODUCT**	**HYDROXOCOBALAMINE ACETATE – VIT. B12**
**LOT.**	A17243E
**APPEARANCE**	Crystalline powder or dark red crystals
**IDENTIFICATION**	Conforms
**A525/A531**	0.33
**A274/A351**	0.8
**pH**	6.0
**LOSS ON WEIGHT**	11.1%
**SUM OF IMPURITY**	<5.0%
**SPECTRUMPHOTOMETRIC ASSAY**	97.5% sps
**ACETONE (GC)**	738 ppm
**METHANOL (GC)**	<1000 ppm
**HEAVY METALS**	Conforms
**TOTAL PLATE COUNT**	<1000 cfu/g
**YEAST & MOLD**	<100 cfu/g
**E. COLI**	Negative
**SALMONELLA**	Negative
**BACTERIAN ENDOTOXINE**	<=0.4 Complies
**CONCLUSION**	This product meets with the specification of Ph. Eur
**COPY CONFORM TO THE ORIGINAL DOCUMENT – VALID ALTHOUGH NOT SIGNED**	

via G. Rossetti 19
20145 Milano (MI)
Italy

CERTIFICATO D'ANALISI<br>CERTIFICATE OF ANALYSIS	
**PRODUCT**	**VITAMIN B5 (D-CALCIUM PANTOTHENATE)**
**LOT.**	16061404
**APPEARANCE**	WHITE OR ALMOST WHITE POWDER
**INFRARED ABSORBATION**	Up to standard figure
**IDENTIFICATION OF CALCIUM ION**	Normal reaction
**pH VALUE**	Conform (6.8-8.0)
**ALKALINITY**	Conform
**SPECIFIC ROTATION**	+26.8°C
**LEAD**	<3 ppm
**ARSENIC**	<1 ppm
**CADMIUM**	<1 ppm
**MERCURY**	<0.1 ppm
**IMPURITY CONTENT**	Conform <1%
**HEAVY METALS**	<=0.002%
**NITROGEN CONTENT**	5.8%
**CALCIUM CONTENT**	8.3%
**LOSS ON DRYING**	2.2%
**RESIDUAL SOLVENT**	0.11%
**TOTAL PLATE COUNT**	<1000 cfu/g
**YEAST & MOLD**	<100 cfu/g
**E. COLI**	Negative/10 g
**SALMONELLA**	Negative/25 g
**ASSAY**	99.2%
**CONCLUSION**	Complies with USP39
**COPY CONFORM TO THE ORIGINAL DOCUMENT – VALID ALTHOUGH NOT SIGNED**	

Meetab
your custom supplements

via G. Rossetti 19
20145 Milano (MI)
Italy

**CERTIFICATO D'ANALISI**<br>CERTIFICATE OF ANALYSIS	
**PRODUCT**	**VITAMIN B2 (Riboflavin)**
**LOT.**	17D10-B11-PPR1717485
**APPEARANCE**	YELLOW- ORANGE POWDER
**IDENTIFICATION**	Conforms
**SPECIFIC ROTATION**	Conforms
**ABSORBANCE (Ph. Eur.)**	Conforms
**LUMIFLAVIN (USP)**	0.0052
**SULPHUR ASH**	0.08/100 g
**RESIDUE ON IGNITION**	0.08%
**LOSS ON DRYING**	1.0g/100g
**ASSAY**	99.9%
**HEAVY METALS**	<10 ppm
**LEAD**	<2 ppm
**ARSENIC**	<1 ppm
**CADMIUM**	<1 ppm
**MERCURY**	<0.1 ppm
**TOTAL PLATE COUNT**	<1000 cfu/g
**YEAST & MOLD**	<100 cfu/g
**E. COLI**	Negative/10 g
**SALMONELLA**	Negative/25 g
**PSUDOMONAS AERUGINOSA**	Negative/g
**STAPHYLOCOCCUS AUREUS**	Negative/g
**COPY CONFORM TO THE ORIGINAL DOCUMENT – VALID ALTHOUGH NOT SIGNED**	

Meetab
your custom supplements

via G. Rossetti 19
20145 Milano (MI)
Italy

**CERTIFICATO D'ANALISI**<br>CERTIFICATE OF ANALYSIS	
**PRODUCT**	**VITAMIN D3 100000 IU/g**
**LOT.**	5431-1712001
**APPEARANCE**	White to off White/Yellowish Powder
**IDENTIFICATION**	Conforms
**ASSAY**	Min. 100000 IU/g
**SIEVE ANALYSIS**	100% Pass 40 Mesh Conforms
**LOSS ON DRYING**	3.91%
**ARSENIC**	Conforms (<0.5 ppm)
**LEAD**	Conforms (0.5 ppm)
**CADMIUM**	Conforms (<0.1 ppm)
**MERCURY**	Conforms (<0.1 ppm)
**TOTAL PLATE COUNT**	10 cfu/g (<1000 cfu/g)
**YEAST & MOLD**	10 cfu/g (<100 cfu/g)
**E. COLI**	Negative
**SALMONELLA**	Negative
**STAPHYLOCOCCUS AUREUS**	Negative
**COLIFORMS**	Negative
**GMO/BSE/TSE/GLUTEN**	Absent
**COPY CONFORM TO THE ORIGINAL DOCUMENT – VALID ALTHOUGH NOT SIGNED**	

via G. Rossetti 19
20145 Milano (MI)
Italy

CERTIFICATO D'ANALISI<br>CERTIFICATE OF ANALYSIS	
**PRODUCT**	**D-BIOTIN (VIT H)**
**LOT.**	VH20160702C
**APPEARANCE**	White or off white crystalline powder
**SOLUBILITY**	Conforms
**IDENTIFICATION**	Conforms
**CLEAR AND COLOUR**	Conforms
**HEAVY METALS**	<10 ppm
**LEAD**	<2 ppm
**SULPHATED ASH**	0.07%
**SPECIFIC OPTICAL ROTATION**	90.7°
**MELTING POINT**	Conforms
**ORGANIC VOLATILE IMPURITY**	Conforms
**ASSAY**	99.9%
**RELATED SUBSTANCES**	Conforms
**LOSS ON DRYNG**	0.08%
**TOTAL PLATE COUNT**	<1000 cfu/g
**YEAST & MOLD**	<100 cfu/g
**E. COLI**	Negative
**SALMONELLA**	Negative
**CONCLUSION**	Confroms to USP38/EP8
**COPY CONFORM TO THE ORIGINAL DOCUMENT – VALID ALTHOUGH NOT SIGNED**	

Meetab
your custom
supplements

via G. Rossetti 19
20145 Milano (MI)
Italy

**CERTIFICATO D'ANALISI**<br>CERTIFICATE OF ANALYSIS	
**PRODUCT**	**(6S)-5-METHYLTETRAHYDROFOLIC ACID, GLUCOSAMINE SALT (QUATREFOLIC®)**
**LOT.**	0001701113
**APPEARANCE**	Creamy to light brown powder
**MOLECULAR FORMULA**	C20H23N7O6(C6H14NO5)2
**MOLECULAR WEIGHT**	817.80
**IDENTIFICATION (IR)**	Conforms
**WATER CONTENT (K.F.)**	<=8.0%
**GLUCOSAMINE ASSAY ON D.B. (HPLC)**	34-36%
**5-METHYLTETRAHYDROFOLIC ACID ASSAY ON D.B. (HPLC)**	54-59%
**TOTAL IMPURITIES**	<=2.5%
**LEAD**	<=0.3 ppm
**ARSENIC**	<=1.5 ppm
**MERCURY**	<=0.1 ppm
**CADMIUM**	<=0.5 ppm
**BORON**	<=10 ppm
**TOTAL PLATE COUNT**	<100 cfu/g
**YEAST & MOLD**	<100 cfu/g
**E. COLI**	Negative/10 g
**SALMONELLA**	Negative 25/g
**ASSAY (ON DRIED BASIS)**	99.0%
**COPY CONFORM TO THE ORIGINAL DOCUMENT – VALID ALTHOUGH NOT SIGNED**	

via G. Rossetti 19
20145 Milano (MI)
Italy

**CERTIFICATO D'ANALISI**<br>CERTIFICATE OF ANALYSIS	
**PRODUCT**	**L-GLUTAMINE KYOWA QUALITY**
**LOT.**	GM-PL-17462
**APPEARANCE**	White Crystalline Powder
**IDENTIFICATION**	Conforms
**STATE OF SOLUTION**	NLT 99.2%
**pH**	5.1
**SPECIFIC ROTATION (AT 20°C)**	+7.0
**CHLORIDE**	NMT 0.020%
**SULFATE**	NMT 0.020%
**IRON**	NMT 100 ppm
**HAVY METALS**	NMT 5 ppm
**LEAD**	NMT 5 ppm
**ARSENIC**	NMT 1 ppm
**FOREIGN AMINO ACID**	NMT 0.5%
**LOSS ON DRYING**	0.01%
**RESIDUE OF IGNITION**	0.01%
**ASSAY (DRIED BASIS)**	99.5%
**TOTAL COUNT (CFU)**	NMT 1.000/g
**YEST AND MOLDS (CFU)**	NMT 100/g
**COLIFORM**	NEG
**INSOLUBLE FOREIGN MATTER**	Conforms
**COPY CONFORM TO THE ORIGINAL DOCUMENT – VALID ALTHOUGH NOT SIGNED**	

via G. Rossetti 19
20145 Milano (MI)
Italy

CERTIFICATO D'ANALISI<br>CERTIFICATE OF ANALYSIS	
**PRODOTTO**	**Amino acid Mix:** L-Lysine HCl, L-Proline, L-Glycine, N-acetyl-L-Cysteine, L-Arginine, L-Methionine.
**LOT.**	201217
**APPEARANCE**	White crystalline powder or colorless crystals
**STATE OF SOLUTION**	>95%
**CHLORIDE**	<=0.02%
**AMMONIUM**	<=0.02%
**SULFATE**	<=0.02%
**MERCURY**	<0.1 ppm
**LEAD**	< 5 ppm
**ARSENIC**	<1 ppm
**CADMIUM**	< 1 ppm
**HEAVY METALS**	<= 10 ppm
**LOSS ON DRYING**	<=0.2%
**RESIDUE ON IGNITION**	<=0.10%
**TOTAL PLATE COUNT**	<1000 cfu/g CONFORM
**YEAST AND MOLDS**	<100 cfu/g CONFORM
**E. COLI**	Negative cfu/g
**SALMONELLA**	Negative/25 g
**ASSAY**	99.5%
**CONCLUSION**	Product conform to USP36/USP/24FCC/AJI92/JP15
**COPY CONFORM TO THE ORIGINAL DOCUMENT(s) – VALID ALTHOUGH NOT SIGNED**	

Meetab
your custom supplements

via G. Rossetti 19
20145 Milano (MI)
Italy

**CERTIFICATO D'ANALISI**<br>CERTIFICATE OF ANALYSIS	
**PRODOTTO**	**OXXYNEA® (MIX SOLUBILE DI ESTRATTI DA FRUTTA E VERDURA)**
**LOT.**	OXFP170316
**TIPO DI PREPARAZIONE UTILIZZATA**	Polvere ad alto potere antiossidante ottenuta da una miscela di frutti e verdure standardizzata in valore ORAC (1 g = 3500 valori ORAC)
**ORIGINE**	France, Spain, Italy
**ASPECT**	Fine powder
**COLOUR**	Brown
**TASTE**	Characteristic
**ODOR**	Typical
**COMPOSITION**	Estratto da frutta 40-50%: Semi d'uva (rossa, bianca), Arancia, Pompelmo, Papaia, Ananas, Fragole, Ciliegie, Mela, Albicocca, Mirtillo, Ribes nero.<br>Estratto da vegetali: 30-40%: Pomodoro, Carote, Cocomero, Tè verde, Broccoli, Cavolo, Cipolla, Aglio, Asparago, Olive, Cetriolo.<br>Maltodestrina 10-20%
**TOTAL POLYPHENOLS (% CATECHINE EQ.)**	83.41%
**ORAC VALUE (µmol TE/g)**	11773.1%
**LOSS ON DRYING %**	0.1%
**ASH %**	1.5%
**SOLUBILITY (1% W/V)**	CONFORMS
**Ph (10% W/V)**	4.4
**BULK DENSITY**	0.50
**TAPPED DENSITY**	0.70
**MESH SIZE (US mesh)**	CONFORMS
**ARSENIC (As)**	<0.1 ppm
**CADMIUM (Cd)**	0.01% ppm
**MERCURY (Hg)**	<0.005 ppm
**LEAD (Pb)**	0.06 ppm
**TOTAL PLATE COUNT**	<1000 cfu/g CONFORM
**YEAST AND MOLDS**	<100 cfu/g CONFORM
**COLIFORMS**	Negative cfu/1 g
**SALMONELLA**	Negative/25 g
**STAPHYLOCOCCUS AUREUS**	Negative cfu/1 g
**COPY CONFORM TO THE ORIGINAL DOCUMENT – VALID ALTHOUGH NOT SIGNED**	

via G. Rossetti 19
20145 Milano (MI)
Italy

**CERTIFICATO D'ANALISI**<br>CERTIFICATE OF ANALYSIS	
**PRODUCT NAME**	**BROCCOLI 1:25**
**LOT.**	NUT/16/0896
**BOTANICAL NAME**	Brassica oleracea L.
**EXTRACTION SOLVENT**	Water
**E/D RATIO**	1:25
**EXCIPIENT**	Maltodextrin from maize
**AUXILIARY SUBSTANCE**	<=0.5% colloidal anydrous silica
**PREPARATION TYPE**	Dry extract
**PART OF PLANT USED**	Leaves
**ORIGIN**	Italy
**ASPECT**	Powder
**COLOUR**	Brownish to greenish
**TASTE**	Characteristic
**ODOR**	Characteristic
**IDENTIFICATION**	Complies (met. TLC)
**PARTICLE SIZE**	>=90% through 35 Mesh (500 micron)
**DENSITY**	0.5 g/ml ~
**LOSS ON DRYING**	<=5%
**HEAVY METALS**	<10 ppm<br>Pb <3 ppm; Cd <1 ppm; Hg <0.1 ppm
**RESIDUAL SOLVENTS**	Cpmplies to Directive 2009/32/EC
**PESTICIDES**	Complies to Reg. 2008/839/CE
**POLYCYCLIC AROMATIC HYDROCARBONS**	Conforms to Reg. UE 1933/2015
**AFLATOXINS**	Aflatoxin B1: <5 ppb<br>Total aflatoxin (B1,B2,G1,G2): <10 ppb
**BACTERIAL COUNT (TAMC)**	<= 5x10000 cfu/g
**YEAST AND MOUDS (TYMC)**	<= 5x10 cfu/g
**PATHOGENS**	Salmonella: absent/25g<br>E. Coli: absent/1 g
**ENTEROBACTERIACEE**	<=100 cfu/g
**GLUTEN FREE**	Yes
**COPY CONFORM TO THE ORIGINAL DOCUMENT – VALID ALTHOUGH NOT SIGNED**	

via G. Rossetti 19
20145 Milano (MI)
Italy

**CERTIFICATO D'ANALISI**<br>CERTIFICATE OF ANALYSIS	
**PRODUCT**	**BITARTRATE CHOLINE**
**LOT.**	3012864
**APPEARANCE**	WHITE CRYSTALLINE POWDER
**IDENTIFICATION**	COMPLIES (IR, B)
**SPECIFIC ROTATION**	+17.9°
**pH (10%)**	3.4
**WATER**	0.07%
**RESIDUE ON IGNITION**	<0.1%
**AS**	<2 ppm
**LEAD**	<0.3 ppm
**TOTAL AMINES**	4.1 ppm
**CROMATOGRAPHIC PURITY**	Conforms
**O.V.I.**	Conforms
**1.4 DIOXANE**	Conforms
**HEAVY METAL**	<10 ppm
**ASSAY (anhydrous basis)**	99.52%
**TOTAL AEROBIC COUNT**	<1000 cfu/g
**YEAST & MOLD**	<100 cfu/g
**E. COLI**	Negative/10 g
**SALMONELLA**	Negative/25 g
**CONFORM**	USP/DAB
**COPY CONFORM TO THE ORIGINAL DOCUMENT – VALID ALTHOUGH NOT SIGNED**	

via G. Rossetti 19
20145 Milano (MI)
Italy

**CERTIFICATO D'ANALISI**<br>CERTIFICATE OF ANALYSIS	
**PRODUCT**	**INOSITOL**
**LOT.**	HZ-IN1702065
**APPEARANCE**	WHITE CRYSTALLINE POWDER
**IDENTIFICATION**	Positive reaction (A,B)
**MELTING POINT**	225.3-226.4°C
**LOSS ON DRYING**	0.02%
**RESIDUE ON IGNITION**	0.02%
**CLARITY/COLOR OF SOLUTION**	meet the requirement
**CHLORIDE**	<0.005%
**SULFATE**	<0.006%
**CALCIUM**	meet the requirement
**HEAVY METALS**	<5 ppm
**LEAD**	<0.5 ppm
**ARSENIC**	<0.5 ppm
**CADMIUM**	<0.5 ppm
**MERCURY**	<0.1 ppm
**TOTAL PLATE COUNT**	<10 cfu/g
**YEAST & MOLD**	<10 cfu/g
**E. COLI**	Negative/10 g
**SALMONELLA**	Negative/25 g
**ASSAY**	98.59%
**CONCLUSION**	The good are complied with FCCVIX/USP38/NF33
**COPY CONFORM TO THE ORIGINAL DOCUMENT – VALID ALTHOUGH NOT SIGNED**	

Meetab
your custom supplements

via G. Rossetti 19
20145 Milano (MI)
Italy

**CERTIFICATO D'ANALISI**<br>CERTIFICATE OF ANALYSIS	
**PRODUCT**	**PARA-AMINOBENZOIC ACID**
**LOT.**	17E15-B09
**APPEARANCE**	WHITE CRYSTALLINE POWDER
**IDENTIFICATION**	Conforms
**PARTICLE SIZE (MESH)**	Conforms
**ASSAY (ON DRIED BASIS)**	99.75%
**RESIDUE ON IGNITION**	0.01%
**LOSS ON DRYING**	0.08%
**HUMIDITY**	1.1%
**HEAVY METALS**	<20 ppm
**TOTAL PLATE COUNT**	<1000 cfu/g
**MERCURY**	<0.1 ppm
**CADMIUM**	<1 ppm
**YEAST & MOLD**	<100 cfu/g
**E. COLI**	Negative/1g
**SALMONELLA**	Negative/25g
**CHEMICAL FORMULA**	C7H7N02
**COPY CONFORM TO THE ORIGINAL DOCUMENT – VALID ALTHOUGH NOT SIGNED**	

Meetab
your custom supplements

via G. Rossetti 19
20145 Milano (MI)
Italy

**CERTIFICATO D'ANALISI**<br>CERTIFICATE OF ANALYSIS	
**PRODOTTO**	**CITRUS BIOFLAVONOIDS 60%**
**LOT.**	201711016
**EXTRACT RATIO**	1 g Extract to 5-7 g Herbs
**SOURCE**	Fruit of the Citrus Aurantium L.
**APPEARANCE**	Fine powder
**COLOUR**	Light brown
**AROMA**	Characteristic
**FLOVOUR**	Characteristic
**IDENTIFICATION**	Positive
**ASSAY BIOFLAVONOID**	61.2%
**SIEVE ANALYSIS**	98% Pass 80 Mesh
**LOSS ON DRYING**	1.4%
**ASH**	0.4%
**BULK DENSITY**	0.41 g/ml
**HEAVY METAL**	< 10 ppm
**ARSENIC**	<1 ppm
**LEAD**	<1 ppm
**CADMIUM**	<1 ppm
**MERCURY**	<0.1 ppm
**SULPHATE**	<5 ppm
**TOTAL PLATE COUNT**	20 cfu/g
**YEAST AND MOULDS**	20 cfu/g
**E.COLI**	Negative
**SALMONELLA**	Negative
**STAPHYLOCOCCUS AUREUS**	Negative
**COLIFORMS**	Max 30 cfu/g Conforms
**GMO STATUS**	Non-GMO
**IRRADIATION STATUS**	Non Irradiated
**BSE/TSE STATUS**	BSE/TSE Free
**SUITABLE FOR VEGETARIAN**	YES
**SUITABLE FOR VEGANS**	YES
**COPY CONFORM TO THE ORIGINAL DOCUMENT – VALID ALTHOUGH NOT SIGNED**	

Meetab
your custom supplements

via G. Rossetti 19
20145 Milano (MI)
Italy

**CERTIFICATO D'ANALISI**<br>CERTIFICATE OF ANALYSIS	
**PRODOTTO**	**PINE BARK EXTRACT**
**LOT.**	20181020
**DESCRIPTION**	Fine powder brown/reddish
**ODOR**	Characteristic
**DENSITY**	500 g/l
**TASTE**	Characteristic
**IDENTIFICATION**	Positive (TLC)
**LIPOSOLUBILITY**	Partially liposoluble
**EXSTACTION SOLVENT**	water/ethanol
**RESIDUAL SOLVENT**	Conform to Reg (CE) 32/2009
**HEAVY METAL**	Pb<3 ppm; Cd<1ppm; Hg<0.1 ppm
**YEAST AND MOULDS**	Conforms (<5*100 ufc/g)
**TOTAL PLATE COUNT**	Conforms (<5*10000 ufc/g)
**E.COLI**	Negative
**SALMONELLA**	Negative
**TOTAL SOLIDS**	3.72%
**ASSAY**	90.8% pycnogenol
**GMO STATUS**	Free
**BSE/TSE STATUS**	Free
**AFLATOXIN**	<10 ppb
**CONFORM**	USP/FCC/EP
**COPY CONFORM TO THE ORIGINAL DOCUMENT – VALID ALTHOUGH NOT SIGNED**	

via G. Rossetti 19
20145 Milano (MI)
Italy

CERTIFICATO D'ANALISI<br>CERTIFICATE OF ANALYSIS	
**PRODOTTO**	**ASTAXANTHIN 5%**
**LOT.**	20171103F
**APPEARANCE**	Powder
**COLOUR**	Dark red
**AROMA**	Characteristic
**FLAVOUR**	Characteristic
**IDENTIFICATION**	Positive
**ASSAY (as dried substance)**	5. 23% Astaxanthin
**SIEVE ANALYSIS**	Conforms
**ASH**	4.63%
**LOSS ON DRYING**	0.98%
**LEAD (Pb)**	Max 3 ppm
**CADMIUM (Cd)**	Max. 1 ppm
**ARSENIC (As)**	Max 1 ppm
**MERCURY (Hg)**	Max 0.1 ppm
**TOTAL PLATE COUNT**	360 cfu/g
**YEAST AND MOULDS**	5 cfu/g
**SALMONELLA**	Negative
**STAPHYLOCOCCUS AUREUS**	Negative
**COLIFORMS**	Conforms
**SHIGELLA**	Negative
**GMO STATUS**	Non-GMO
**IRRADIATION STATUS**	Non Irradiated
**BSE/TSE STATUS**	BSE/TSE Free
**CONFORM**	USP/FCC/EP/FDA
**COPY CONFORM TO THE ORIGINAL DOCUMENT – VALID ALTHOUGH NOT SIGNED**	

via G. Rossetti 19
20145 Milano (MI)
Italy

**CERTIFICATO D'ANALISI**<br>CERTIFICATE OF ANALYSIS	
**PRODOTTO**	**LUTEIN 5%**
**LOT.**	MAE-170701
**APPEARANCE**	Fine Powder
**COLOUR**	Orange Yellow
**AROMA**	Characteristic
**FLAVOUR**	Characteristic
**IDENTIFICATION**	Positive
**ASSAY (as dried substance)**	5.19 % Lutein
**SIEVE ANALYSIS**	Conforms
**ASH**	0.66%
**LOSS ON DRYING**	0.64%
**HEAVY METALS**	Max. 5 ppm
**LEAD (Pb)**	Max 1 ppm
**CADMIUM (Cd)**	Max. 2 ppm
**ARSENIC (As)**	Max 2 ppm
**MERCURY (Hg)**	Max 0.1 ppm
**TOTAL PLATE COUNT**	<1000 cfu/g
**YEAST AND MOULDS**	<100 cfu/g
**E. COLI**	Negative
**SALMONELLA**	Negative
**STAPHYLOCOCCUS AUREUS**	Negative
**GMO STATUS**	Non-GMO
**IRRADIATION STATUS**	Non Irradiated
**BSE/TSE STATUS**	BSE/TSE Free
**CONFORM**	USP/FCC/EP/FDA
**COPY CONFORM TO THE ORIGINAL DOCUMENT – VALID ALTHOUGH NOT SIGNED**	

via G. Rossetti 19
20145 Milano (MI)
Italy

**CERTIFICATO D'ANALISI**<br>CERTIFICATE OF ANALYSIS	
**PRODOTTO**	**ALPHA LIPOIC ACID**
**LOT.**	ALA.G.1801101
**APPEARANCE**	Granular
**COLOUR**	Yellowish
**AROMA**	Characteristic
**FLAVOUR**	Characteristic
**IDENTIFICATION**	Positive
**ASSAY (as dried substance)**	99.3%
**MELTING POINT**	61.54°C
**SPECIFIC ROTATION**	0°
**SIEVE ANALYSIS**	Conforms
**ASH**	0.04%
**LOSS ON DRYING**	0.13%
**PARTICLE SIZE**	Conforms
**BULK DENSITY**	0.50 g/ml
**TAPPED DENSITY**	Conforms
**HEAVY METALS**	Max. 10 ppm
**LEAD (Pb)**	Max 3 ppm
**CADMIUM (Cd)**	Max. 1 ppm
**ARSENIC (As)**	Max 1 ppm
**MERCURY (Hg)**	Max 0.1 ppm
**TOTAL PLATE COUNT**	<1000 cfu/g
**YEAST AND MOULDS**	<100 cfu/g
**E. COLI**	Negative
**SALMONELLA**	Negative
**STAPHYLOCOCCUS AUREUS**	Negative
**SINGLE IMPURITY**	0.07%
**TOTAL IMPURITIES**	0.016%
**LIMIT OF POLYMER**	Conforms to USP
**GMO STATUS**	Non-GMO
**IRRADIATION STATUS**	Non Irradiated
**BSE/TSE STATUS**	BSE/TSE Free
**CONFORM**	USP/FCC/EP/FDA
**COPY CONFORM TO THE ORIGINAL DOCUMENT – VALID ALTHOUGH NOT SIGNED**	

via G. Rossetti 19
20145 Milano (MI)
Italy

CERTIFICATO D'ANALISI<br>CERTIFICATE OF ANALYSIS	
**PRODOTTO**	**ACEROLA 25% VIT. C (Malpighia punicifolia L.) fruit**
**LOT.**	L100181L
**APPEARANCE**	Rosy fine powder, odour characteristic
**LOSS ON DRYING**	< 5%
**ASH**	< 15%
**ASSAY**	<25% Vit. C
**LEAD (Pb)**	Max 3 ppm
**CADMIUM (Cd)**	Max. 1 ppm
**ARSENIC (As)**	Max 1 ppm
**MERCURY (Hg)**	Max 0.1 ppm
**AFLATOXIN Bb1,B2,G1,G2**	< 10 PPB
**PARTICLE SIZE**	Conforms
**BULK DENSITY**	600-700 g/L
**TOTAL PLATE COUNT**	<5*10e4 cfu/g
**YEAST AND MOULDS**	<5*10e2 cfu/g
**E. COLI**	Negative
**SALMONELLA**	Negative
**GMO STATUS**	Non-GMO
**IRRADIATION STATUS**	Non Irradiated
**BSE/TSE STATUS**	BSE/TSE Free
**CONFORM**	USP/FCC/EP/FDA
**COPY CONFORM TO THE ORIGINAL DOCUMENT – VALID ALTHOUGH NOT SIGNED**	

Meetab
your custom supplements

via G. Rossetti 19
20145 Milano (MI)
Italy

**CERTIFICATO D'ANALISI**<br>CERTIFICATE OF ANALYSIS	
**PRODOTTO**	**RESVERATROL 98%**
**LOT.**	NT180402003
**APPEARANCE**	Fine Powder
**COLOUR**	White
**AROMA**	Characteristic
**FLAVOUR**	Characteristic
**IDENTIFICATION**	Positive
**ASSAY**	98.81% Resveratrol
**SIEVE ANALYSIS**	Conforms
**ASH**	0.26%
**LOSS ON DRYING**	0.19%
**HEAVY METALS**	Max. 10 ppm
**LEAD (Pb)**	Max 3 ppm
**CADMIUM (Cd)**	Max. 1 ppm
**ARSENIC (As)**	Max 1 ppm
**MERCURY (Hg)**	Max 0.1 ppm
**TOTAL PLATE COUNT**	<1000 cfu/g
**YEAST AND MOULDS**	<100 cfu/g
**E. COLI**	Negative
**SALMONELLA**	Negative
**STAPHYLOCOCCUS AUREUS**	Negative
**AFLATOXIN Bb1,B2,G1,G2**	< 10 PPB
**GMO STATUS**	Non-GMO
**IRRADIATION STATUS**	Non Irradiated
**BSE/TSE STATUS**	BSE/TSE Free
**CONFORM**	USP/FCC/EP/FDA
**COPY CONFORM TO THE ORIGINAL DOCUMENT – VALID ALTHOUGH NOT SIGNED**	

Meetab
your custom supplements

via G. Rossetti 19
20145 Milano (MI)
Italy

**CERTIFICATO D'ANALISI**<br>CERTIFICATE OF ANALYSIS	
**PRODOTTO**	**ASCORBYL PALMITATE**
**LOT.**	10500120180103
**APPEARANCE**	Fine powder Light white-yellow
**IDENTIFICATION**	Positive
**ASPECT**	Conform
**MELTING POINT**	CONFORM (ABOUT 115°C)
**SPECIFIC ROTATION**	23.4°
**LOSS ON DRYING**	0.18%
**PERDITA ALLA CALCINAZIONE**	0.05%
**HEAVY METAL**	< 10 ppm
**ARSENIC**	<1 ppm
**LEAD**	<0.1 ppm
**MERCURY**	<0.1 ppm
**CADMIUM**	<1 ppm
**TOTAL PLATE COUNT**	<100 cfu/g
**YEAST AND MOULDS**	<10 cfu/g
**E.COLI**	Negative
**SALMONELLA**	Negative
**STAPHYLOCOCCUS AUREUS**	Negative
**COLIFORMS**	Max 30 cfu/g Conforms
**RESIDUAL SOLVENT**	<0.10%
**ASSAY (USP-FCC)**	98.38%
**GMO STATUS**	Non-GMO
**IRRADIATION STATUS**	Non Irradiated
**BSE/TSE STATUS**	BSE/TSE Free
**CONFORM**	USP/FCC/EP
**COPY CONFORM TO THE ORIGINAL DOCUMENT – VALID ALTHOUGH NOT SIGNED**	

Meetab
your custom supplements

via G. Rossetti 19
20145 Milano (MI)
Italy

**CERTIFICATO D'ANALISI**<br>CERTIFICATE OF ANALYSIS	
**PRODOTTO**	**CALCIUM ASCORBATE**
**LOT.**	31703015
**APPEARANCE**	Fine powder Light white
**IDENTIFICATION**	Positive
**ASPECT**	Conform
**pH (sol.10%)**	7.3
**SPECIFIC ROTATION**	+96.2°
**LOSS ON DRYING**	0.05%
**HEAVY METAL**	< 10 ppm
**FLUORIDE**	< 10 ppm
**OXALATES**	Conforms
**ARSENIC**	<1 ppm
**LEAD**	<2 ppm
**MERCURY**	<0.1 ppm
**CADMIUM**	<1 ppm
**TOTAL PLATE COUNT**	<1000 cfu/g
**YEAST AND MOULDS**	20 cfu/g
**E.COLI**	Negative
**SALMONELLA**	Negative
**STAPHYLOCOCCUS AUREUS**	Negative
**COLIFORMS**	Max 30 cfu/g Conforms
**RESIDUAL SOLVENT**	<3000 ppm
**GMO STATUS**	Non-GMO
**IRRADIATION STATUS**	Non Irradiated
**BSE/TSE STATUS**	BSE/TSE Free
**ASSAY (anhydrous basis)**	99.5%
**CONFORM**	USP/FCC
**COPY CONFORM TO THE ORIGINAL DOCUMENT – VALID ALTHOUGH NOT SIGNED**	

Meetab
your custom supplements

via G. Rossetti 19
20145 Milano (MI)
Italy

CERTIFICATO D'ANALISI<br>CERTIFICATE OF ANALYSIS	
**PRODOTTO**	**GREEN TEA EXTRAXT 95% POLYPHENOLS**
**LOT.**	11722
**APPEARANCE**	Powder
**COLOUR**	Light yellow to reddish brown
**AROMA**	Characteristic
**FLAVOUR**	Characteristic
**IDENTIFICATION**	Positive
**ASSAY**	95% polyphenols, 40% EGCG
**SIEVE ANALYSIS**	Conforms
**ASH**	Max 2%
**LOSS ON DRYING**	Max 6%
**HEAVY METALS**	Max. 10 ppm
**LEAD (Pb)**	Max 3 ppm
**CADMIUM (Cd)**	Max. 1 ppm
**ARSENIC (As)**	Max 2 ppm
**MERCURY (Hg)**	Max 0.1 ppm
**TOTAL PLATE COUNT**	<10000 cfu/g
**YEAST AND MOULDS**	<100 cfu/g
**E. COLI**	Negative
**SALMONELLA**	Negative
**STAPHYLOCOCCUS AUREUS**	Negative
**AFLATOXIN Bb1,B2,G1,G2**	< 10 PPB
**GMO STATUS**	Non-GMO
**IRRADIATION STATUS**	Non Irradiated
**BSE/TSE STATUS**	BSE/TSE Free
**CONFORM**	USP/FCC/EP/FDA
**COPY CONFORM TO THE ORIGINAL DOCUMENT – VALID ALTHOUGH NOT SIGNED**	

Meetab
your custom supplements

via G. Rossetti 19
20145 Milano (MI)
Italy

**CERTIFICATO D'ANALISI**<br>CERTIFICATE OF ANALYSIS	
**PRODOTTO**	**QUERCETIN (SOPHORA JAPONICA L.) SEED**
**LOT.**	NT1800705003
**APPEARANCE**	Crystalline Powder
**COLOUR**	yellow – green
**AROMA**	Characteristic
**FLAVOUR**	Characteristic
**IDENTIFICATION**	Positive
**ASSAY**	98.17% quercitin
**SIEVE ANALYSIS**	Conforms
**ASH**	0.16%
**LOSS ON DRYING**	9.93%
**HEAVY METALS**	Max. 10 ppm
**LEAD (Pb)**	Max 3 ppm
**CADMIUM (Cd)**	Max. 1 ppm
**ARSENIC (As)**	Max 1 ppm
**MERCURY (Hg)**	Max 0.1 ppm
**TOTAL PLATE COUNT**	<1000 cfu/g
**YEAST AND MOULDS**	<100 cfu/g
**E. COLI**	Negative
**SALMONELLA**	Negative
**STAPHYLOCOCCUS AUREUS**	Negative
**AFLATOXIN Bb1,B2,G1,G2**	< 4 ppb
**GMO STATUS**	Non-GMO
**IRRADIATION STATUS**	Non Irradiated
**BSE/TSE STATUS**	BSE/TSE Free
**CONFORM**	USP/FCC/EP/FDA
**COPY CONFORM TO THE ORIGINAL DOCUMENT – VALID ALTHOUGH NOT SIGNED**	

Meetab
your custom supplements

via G. Rossetti 19
20145 Milano (MI)
Italy

CERTIFICATO D'ANALISI<br>CERTIFICATE OF ANALYSIS	
**PRODOTTO**	**TART CHERRY (PRUNUS CERASUS EXTRACT 4:1)**
**LOT.**	NT180630001
**APPEARANCE**	Fine Powder
**COLOUR**	Brown
**AROMA**	Characteristic
**FLAVOUR**	Characteristic
**IDENTIFICATION**	Positive
**ASSAY**	04:01
**SIEVE ANALYSIS**	Conforms
**ASH**	3.26%
**LOSS ON DRYING**	3.98%
**HEAVY METALS**	Max. 10 ppm
**LEAD (Pb)**	Max 3 ppm
**CADMIUM (Cd)**	Max. 1 ppm
**ARSENIC (As)**	Max 1 ppm
**MERCURY (Hg)**	Max 0.1 ppm
**TOTAL PLATE COUNT**	<1000 cfu/g
**YEAST AND MOULDS**	<100 cfu/g
**E. COLI**	Negative
**SALMONELLA**	Negative
**STAPHYLOCOCCUS AUREUS**	Negative
**AFLATOXIN Bb1,B2,G1,G2**	< 4 PPB
**GMO STATUS**	Non-GMO
**IRRADIATION STATUS**	Non Irradiated
**BSE/TSE STATUS**	BSE/TSE Free
**CONFORM**	USP/FCC/EP/FDA
**COPY CONFORM TO THE ORIGINAL DOCUMENT – VALID ALTHOUGH NOT SIGNED**	

via G. Rossetti 19
20145 Milano (MI)
Italy

**CERTIFICATO D'ANALISI**<br>CERTIFICATE OF ANALYSIS	
**PRODOTTO**	**BROMELIN 2500 GDU/g (ANANAS COMOSUS L. MERR)**
**LOT.**	B01/CB4/18
**APPEARANCE**	Powder
**COLOUR**	White
**AROMA**	Characteristic
**FLAVOUR**	Characteristic
**IDENTIFICATION**	Positive
**ASSAY**	2521 GDU/g
**SIEVE ANALYSIS**	Conforms
**ASH**	Conform
**LOSS ON DRYING**	3.9%
**HEAVY METALS**	Max. 10 ppm
**LEAD (Pb)**	Max 3 ppm
**CADMIUM (Cd)**	Max. 1 ppm
**ARSENIC (As)**	Max 1 ppm
**MERCURY (Hg)**	Max 0.1 ppm
**TOTAL PLATE COUNT**	<1000 cfu/g
**YEAST AND MOULDS**	<100 cfu/g
**E. COLI**	Negative
**SALMONELLA**	Negative
**STAPHYLOCOCCUS AUREUS**	Negative
**AFLATOXIN Bb1,B2,G1,G2**	< 10 PPB
**GMO STATUS**	Non-GMO
**IRRADIATION STATUS**	Non Irradiated
**BSE/TSE STATUS**	BSE/TSE Free
**CONFORM**	USP/FCC/EP/FDA
**COPY CONFORM TO THE ORIGINAL DOCUMENT – VALID ALTHOUGH NOT SIGNED**	

MEETAB s.r.l.
via G. Rossetti 19 - 20145 - Milano (MI)
P.IVA: 07790350966

NATUR-FARMA S.R.L.
Via Mazzini 29/F 37040 Pressana (VR)
C.F / P.Iva : 04022190237
info@naturfarma.it Tel.0442 411196
22/01/19

Certificate of analsyis
Certificado de analisis

VIT. E

D-alpha tocopheryl acetate (700 UI-g) (VIT E Nat)
EP 8.0

Batch	Manufactured	Expiration
CP20170701	01/07/17	30/06/19

Characters	Almost white, yellowish or light brown, small particles.	Conform	EP
Identification:			
Thin-layer chromatography	The principal spot in the chromatogram obtained with the test solution is similar in position and size to the principal spot in the chromatogram obtained with the reference solution.	Positive	EP 2.2.27
Retention time	The principal peak in the chromatogram obtained with the test solution is similar in retention time and size to the principal peak in the chromatogram obtained with reference solution.	Confom	EP
Assay	≥51.5%	54.6%	EP 2.2.28
Assay	≥700IU	742IU	EP 2.2.28
Loss on Drying	≤5.0%	1.4%	EP 2.2.23
Bulk Density	0.45 g/cm(3)~0.60 g/cm(3)	0.45 g/cm(3)	EP 2.9.34
Particle Size	Residue on 40 meshes ≤1%	Conform	EP 2.9.38
*Benzo(a)Pyrene	≤2 ppb	< 2ppb	HPLC
*Heavy Metals (as Pb)	≤10ppm	< 10ppm	EP 2.4.8
*Heavy Metals:			
Lead	≤3 mg/kg	Not detected	AAS
Arsenic	≤1 mg/kg	Not detected	AAS
Cadmium	≤1 mg/kg	Not detected	AAS
Mercury	< 0.1 mg/kg	Not detected	AAS
*Microbiology			
Total Bacterial Count	≤1000cfu/g	Conform	EP 2.6.12
Yeast and Molds	≤100cfu/g	Conform	EP 2.6.12
Coliform	< 0.3MPN/g	Negative	EP 2.6.12
Salmonella	Negative/25g	Negative	EP 2.6.13
Staphylococcus Aureus	Negative/10g	Negative	EP 2.6.13

Conclusion: Conform to EP 8.0
Remarks: *Test periodically to ensure requirements are met.
Specification: 700IU

NATUR-FARMA S.R.L.
Via Mazzini 29/F 37040 Pressana (VR)
C.F / P.Iva : 04022190237
info@naturfarma.it Tel.0442 411196

22/01/19

Vit. B6

Document	Revision Status	Page
Specification / Certificate of Analysis	04	1 of 3

Specification	Version	Date	Name	Sign
released QA	0001	29.12.2016	Ellen Biskup	Biskup

Pyridoxal-5-Phosphate			
Quality			
Synonyma	PLP, P5P	Product Code	13572
Chemical Data	C8H10NO6P*H2O; Mr 265.2	Batch-No.	C041804804

Definition
Vitamin B6 derivate

Origin/Synthesis (short description)

Characters	Reference	Requirements	Observations
Appearance	Internal	Powder	Complies
Colour	Internal	Pale yellow	Complies
Solubility	Internal	Sparingly soluble in water, soluble in solutions of alkali hydroxides	Complies

Assay / Ratio	Reference	Requirements	Observations
Assay (calc. on dried substance)	Internal	98.5 % - 101.0 %	99.3 %
Purity (HPLC)	Internal	Min. 99.0 %	99.3 %

Identification	Reference	Requirements	Observations
IR	Internal	Corresponds to reference spectrum	Complies
Melting point	Internal	140.0 °C - 145.0 °C	144.0°

Tests	Reference	Requirements	Observations
Moisture	Internal	Max. 10.0 %	8.7 %
pH (0.25 % in water)	Internal	2.6 - 3.0	2.8
Organic volatile impurities	Internal	Complies to USP28	Complies
Residue organic solvent	Internal	Max. 100 ppm	Complies
Particle size	Internal	Min. 95 % pass 30 mesh	Complies

Certificate of Analysis	Date	Name	Sign
released QA	08.08.2018	Sabine Schwarz	S.Schwarz

NATUR-FARMA S.R.L.
Via Mazzini 29/F 37040 Pressana (VR)
C.F / P.Iva : 04022190237
info@naturfarma.it Tel.0442 411196

22/01/19

VIT B6

Document	Revision Status	Page
**Specification / Certificate of Analysis**	04	2 of 3

Specification	Version	Date	Name	Sign
released QA	0001	29.12.2016	Ellen Biskup	Biskup

Pyridoxal-5-Phosphate			
Quality			
Synonyma	PLP, P5P	Product Code	13572
Chemical Data	C8H10NO6P*H2O; Mr 265.2	Batch-No.	C041804804

Tests	Reference	Requirements	Observations
Bulk density	Internal	Report data	0.45 g/ml
Tapped density	Internal	Report data	0.67 g/ml
Heavy metals	Internal	Max. 10 ppm	Complies
Arsenic	Internal	Max. 2 ppm	0.1 ppm
Lead	Internal	Max. 3 ppm	Not detectable
Mercury	Internal	Max. 0.1 ppm	Not detectable
Cadmium	Internal	Max. 1 ppm	Not detectable

Manufacturing and Release Data	Requirements	Observations
Manufacturing date		03/2018
Analysis date		
Retest Schedule	Min. 24 months	03/2020

Storage	Reference	Requirements
Containers	Internal	Tightly closed, cool, dark and dry

Further remarks
Appended information without quality change; 26.06.2017 Biskup

Regulatory Data (acc. to supplier information and sampling/testing plans within scope of due care)

Not a hazardous substance acc. to EC reg. 1272/2008 and 1907/2006. MSDS not required.
Product has not been subjected to irradiation.
Free of BSE/TSE (Bovine/Transmissible Spongiforme Encephalopathy).
No obligation of GMO labelling as defined in EC reg. 1829/2003 / 1830/2003.
Conform to contaminants reg. (EC) 1881/2006 and amendments as far as applicable.
Free of allergens subject to labelling acc. to EU reg. 1169/2011.
No obligation of nano material labelling as defined in EU reg. 1169/2011

Certificate of Analysis	Date	Name	Sign
released QA	08.08.2018	Sabine Schwarz	S.Schwarz

Document	Revision Status	Page
**Specification / Certificate of Analysis**	04	3 of 3

Specification	Version	Date	Name	Sign
released QA	0001	29.12.2016	Ellen Biskup	Biskup

Pyridoxal-5-Phosphate			
Quality			
Synonyma	PLP, P5P	Product Code	13572
Chemical Data	C8H10NO6P*H2O; Mr 265.2	Batch-No.	C041804804

Regulatory Data
Conforms EC reg. 178/2002 for food raw materials conc. food safety, traceability, hygiene management.
Suitable for vegetarian and vegan food. Produced without the use of products of animal origin.
Without use of palm oil and palm oil derivatives.

Vit B6

NATUR-FARMA S.R.L.
Via Mazzini 29/F 37040 Pressana (VR)
C.F / P.Iva : 04022190237
info@naturfarma.it Tel.0442 411196

22/01/19

Certificate of Analysis	Date	Name	Sign
released QA	08.08.2018	Sabine Schwarz	S.Schwarz

NATUR-FARMA S.R.L.
Via Mazzini 29/F 37040 Pressana (VR)
C.F / P.Iva : 04022190237
info@naturfarma.it Tel.0442 411196
22/01/19

TÈ VERDE E.S.

TÈ VERDE 95% POLIFENOLI 65% CATECHINE

CERTIFICATO DI ANALISI
Codice: LiP00074

Lotto	170326
Data di scadenza	26/03/2020
Nome prodotto	Tè verde 95% Polifenoli 65% Catechine
Nome botanico	Camelia Sinensis (L.) Kuntze
Famiglia botanica	Theaceae
Numero CAS	84650-60-2
Solvente d'estrazione	Acqua, etanolo
Rapporto E/D	1:45/50
Eccipienti	Maltodestina da mais
Sostanze ausiliarie	<= 0.5% silice colloidale anidra
Tipo di preparazione utilizzata	Estratto secco
Parte della pianta usata	Foglie
Origine	Italia, Asia
**DESCRIZIONE**	**RISULTATO**
Aspetto	Polvere fine
Colore	Bruno/Rossastro
Odore	Caratteristico
Sapore	Caratteristico
Identificazione	TLC
Titolo: Polifenoli	98.65 % (metodo spettrofotometrico)
Catechine totali	74.25% (met. HPLC)
EGCG	40.1% (met. HPLC)
Granulometria	Conforme
Densità	0.5 g /ml
Perdita all'essiccamento	3.10 %
Metalli pesanti	< 10 ppm

Data:
17/07/2018

Il presente certificato è conforme a quello del nostro fornitore Le indicazioni sopra riportate non Vi sollevano in ogni caso dall'identificare e controllare il prodotto in relazione all'impiego da Voi previsto.

NATUR-FARMA S.R.L.
Via Mazzini 29/F 37040 Pressana (VR)
C.F / P.Iva : 04022190237
info@naturfarma.it Tel.0442 411196

22/01/19

TÉ VERDE E.S.

Piombo	< 3 ppm
Cadmio	< 1 ppm
Mercurio	< 0.1 ppm
Idrocarburi policiclici aromatici	Conforme al Reg. 1933/2015
Residuo solventi	Conforme alla Dir. 2009/32/CE
Pesticidi	Conforme al Reg. 2008/839 /CE
Aflatossine	Aflatossina B1 : < 5 ppb<br>Aflatossine totali (B1,B2,G1,G2): < 10 ppb
Carica batterica (TAMC)	Conforme
Lieviti e Muffe(TYMC)	Conforme
Patogeni	Salmonella: assente in 25 g<br>Escherichia coli: assente in 1 g
Enterobacteriaceae	< 100 cfu/g
Conservazione	In luogo fresco, asciutto e ventilato, al riparo dalla luce, nei contenitori originali, ben chiusi, o in contenitori di plastica inerte.
Note	Prodotto non contenente OGM (Reg. 1829/2003 - 1830/2003 CE). Prodotto non irradiato. BSE/TSE FREE – Melamina conforme al Reg. Ue 594/2012. NON CONTIENE nessuno degli ingredienti riportati nell'allegato IIIbis della Direttiva 2007/68/CE.
GLUTEN FREE	Si
Certificato Kosher	No
Certificato Hallal	No
Certificato biologico	No
Alimentare	Si

Data:
17/07/2018

Il presente certificato è conforme a quello del nostro fornitore. Le indicazioni sopra riportate non Vi sollevano in ogni caso dall'identificare e controllare il prodotto in relazione all'impiego da Voi previsto.

NATUR-FARMA S.R.L.
Via Mazzini 29/F 37040 Pressana (VR)
C.F / P.Iva : 04022190237
info@naturfarma.it Tel.0442 411196

22/01/19

QUERCETINA

Product and batch Information			
**Product Name:**	Quercetin	**Country of Origin:**	China
**Botanic Name:**	Sophora Japonica L.	**Batch:**	NT180705003
**Piant Part:**	Seed	**Manufacture Date**	JULY 05,2018
**Analysis**	5kg/1 drums	**Analysis Date**	JULY 05,2018
**Solvent extraction**	Water & ethanol	**Excipent**	NONE
**Analysis Item**	**Specification**	**Result**	**Test Method**
	**Physical & Chemical Data**		
Appearance	Crystalline powder	Conform	Organoleptic
Color	Yellow-green	Conform	Visual
Odour	Characteristic	Conform	Organoleptic
	**Analytical Quality**		
Identification	Identical to R.S. sample	Identical	HPTLC
**Assay**	**NLT 98.0%**	**98,17%**	**UV**
Hydrocarbons PAHs	less than 50ppb	Conform	GC-MS
Benzo(a)pyren	≤10 μg/kg	<10 μg/kg	
Sieve Analysis	100% through 80 mesh	Conform	USP39 <786>
Solubility	Practically insoluble in water	Conform	
	Soluble in aqueous alkaline sol.	Conform	
Water (KF)	8%-12%	9,93%	Eur.Ph.7.0 [2.5.12]
Sulphated ash	≤ 0.5%	0,16%	Eur.Ph.7.0 [2.4.16]
	**Contaminants**		
Lead (Pb)	≤ 3,0 mg/kg	Conform	Eur.Ph.7.0<2.2.58>ICP-MS
Arsenic (As)	≤ 1,0 mg/kg	Conform	Eur.Ph.7.0<2.2.58>ICP-MS
Cadmium(Cd)	≤ 1,0 mg/kg	Conform	Eur.Ph.7.0<2.2.58>ICP-MS
Mercury(Hg)	≤ 0.1 mg/kg	Conform	Eur.Ph.7.0<2.2.58>ICP-MS
Heavy metal	≤ 10,0 mg/kg	Conform	Eur.Ph.7.0<2.2.58>ICP-MS
Solvents Residue	Meet Eur.ph.7.0 <5,4> and E.D. 2009/32/EC	Conform	Eur.Ph.7.0<2.4.24>
Pesticides Residues	Meet Eur.ph.7.0 <5,4> and E.D. 2008/839/CE	Conform	Gas Chromatography
	**Microbiological Control**		
Total Plate Count	≤1000 cfu/g	Conform	USP39 <61>
Yeast & Mold	≤100 cfu/g	Conform	USP39 <61>
E.Coli	Negative	Conform	USP39 <62>
Salmonella	Negative	Conform	USP39 <62>
Aflatoxins B1	< 2 μg/ kg	Negative	USP39 <62>
Aflatoxins ∑ B1, B2, G1, G2	< 4 μg/ kg	Negative	USP39 <62>

NATUR-FARMA S.R.L.
Via Mazzini 29/F 37040 Pressana (VR)
C.F / P.Iva : 04022190237
info@naturfarma.it Tel.0442 411196

22/01/19

QUERCETINA

General Status			
Non-irradiation	Conform	Conform	
GMO	FREE	FREE	
BSE/TSE	FREE	FREE	
Melamine	Complies with the Eu reg. 594/2012	Conform	
The products does not contain any of the ingredients listed in Annex II to Directive 1169/11. "			
**Packaging and storage**			
Packing	Pack in paper-drums and two plastic-bags inside N.W. 25 kgs I.D.35xH51cm.		
Storage	Store in a well-closed container away from moisture, light, oxygen.		
Shelf Life	24 months under the conditions above and in its original packaging		
Expiration Date	JULY 04 2020		

- Revisionata il 03/09/2018

NATUR-FARMA [illegible]
Via Mazzini 29/F 37040 Pressana (VR)
C.F / P.Iva : 04022190237
info@naturfarma.it Tel.0442 411196

22/01/19

RESVERATROLO

Product and batch Information			
**Product Name:**	Resveratrol	**Country of Origin:**	China
**Botanic Name:**	Polygonum cuspidatum	**Batch:**	NT180402003
**Piant Part:**	Root	**Manufacture Date**	APR 02,2018
**Analysis**	3Kg/1 drums	**Analysis Date**	APR 02,2018
**Solvent extraction**	Water & ethanol	**Excipent**	None
**Analysis Item**	**Specification**	**Result**	**Test Method**
**Physical & Chemical Data**			
Appearance	Fine powder	Conform	Organoleptic
Color	White	Conform	Visual
Odour	Characteristic	Conform	Organoleptic
**Analytical Quality**			
Identification	Identical to R.S. sample	Identical	HPTLC
**Resveratrol**	**NLT 98.0%**	**98,81%**	**HPLC**
Hydrocarbons PAHs	less than 50ppb	Conform	GC-MS
Benzo(a)pyren	≤10 µg/kg	<10 µg/kg	
Sieve Analysis	100% through 80 mesh	Conform	USP39 <786>
Water (KF)	≤ 5.0%	0,19%	Eur.Ph.7.0 [2.5.12]
Total Ash	≤ 5.0%	0,26%	Eur.Ph.7.0 [2.4.16]
**Contaminants**			
Lead (Pb)	≤ 3,0 mg/kg	Conform	Eur.Ph.7.0<2.2.58>ICP-MS
Arsenic (As)	≤ 1,0 mg/kg	Conform	Eur.Ph.7.0<2.2.58>ICP-MS
Cadmium(Cd)	≤ 1,0 mg/kg	Conform	Eur.Ph.7.0<2.2.58>ICP-MS
Mercury(Hg)	≤ 0.1 mg/kg	Conform	Eur.Ph.7.0<2.2.58>ICP-MS
Heavy metal	≤ 10,0 mg/kg	Conform	Eur.Ph.7.0<2.2.58>ICP-MS
Solvents Residue	Meet Eur.ph.7.0 <5,4> and E.D. 2009/32/EC	Conform	Eur.Ph.7.0<2.4.24>
Pesticides Residues	Meet Eur.ph.7.0 <5,4> and E.D. 2008/839/CE	Conform	Gas Chromatography
**Microbiological Control**			
Total Plate Count	≤1000 cfu/g	Conform	USP39 <61>
Yeast & Mold	≤100 cfu/g	Conform	USP39 <61>
E.Coli	Negative	Conform	USP39 <62>
Salmonella	Negative	Conform	USP39 <62>
Aflatoxins B1	< 2 µg/ kg	Negative	USP39 <62>
Aflatoxins ∑ B1, B2, G1, G2	< 4 µg/ kg	Negative	USP39 <62>

NATUR-FARMA S.R.L.
Via Mazzini 29/F 37040 Pressana (VR)
C.F / P.Iva : 04022190237
info@naturfarma.it Tel.0442 411196

22/01/19

RESVERATROLO

General Status			
Non-irradiation	Conform	Conform	
GMO	FREE	FREE	
BSE/TSE	FREE	FREE	
Melamine	Complies with the Eu reg. 594/2012	Conform	
The products does not contain any of the ingredients listed in Annex II to Directive 1169/11. "			

Packaging and storage	
Packing	Pack in paper-drums and two plastic-bags inside N.W. 25 kgs I.D.35xH51cm.
Storage	Store in a well-closed container away from moisture, light, oxygen.
Shelf Life	24 months under the conditions above and in its original packaging
Expiration Date	APR 01 2020

- Revisionata il 03/09/2018

UR-FARMA S.R.L.
Via Mazzini 29/F 37040 Pressana (VR)
C.F / P.Iva : 04022190237
info@naturfarma.it Tel.0442 411196

22/01/19

N-ACETIL-L-CISTEINA

Prodotto	N-ACETIL-L-CISTEINA (NO A.P.I.)
Codice	012750

SPECIFICHE

Aspetto	Polvere cristallina bianca o quasi
Identificazione IR	Conforme
Titolo(%)	98,5-101,0
Perdita alla calcinazione(ceneri solforiche)(%)	0,2 max.
Cloruri(%)	0,04 max.
Solfati(%)	0,03 max.
Ammonio (%)	0,02 max.
Ferro(ppm)	20 max.
Piombo(ppm)	1 max.
Arsenico(ppm)	0,5 max.
Cadmio (ppm)	0,5 max.
Mercurio (ppm)	0,1 max.
Zinco(ppm)	10 max.
Metalli pesanti(ppm)	10 max.
Impurezze individuali(%)	0,5 max.
Impurezze totali(%)	2,0 max.
Potere rotatorio (°) (20°C)	21,3-27,0
pH	2,0-2,8
Perdita all'essiccamento(%)	0,5 max.
	ANALISI MICROBIOLOGICA
Carica batterica (CFU/g)	1000 max.
Lieviti e muffe/g	100 max.
Enterobatteri/10g	Negativo
Salmonella/10g	Negativo
Coliformi(CFU/g)	Negativo
E.Coli/g	Negativo
Stafilococcus aureus(CFU/g)	Negativo
Bacillus sp./g	Negativo
Clostridium sporogenes (g)	Negativo
Il prodotto è conforme a	EP-USP(ed.correnti)
Il prodotto è conforme a	AJI (ed.corrente)

Data	12/06/2018
Codice	012750

Versione 00 del 12/06/2018

NATUR-FARMA S.R.L.
Via Mazzini 29/F 37040 Pressana (VR)
C.F / P.Iva : 04022190237
info@naturfarma.it Tel.0442 411196
22/01/19

N-ACETIL-L-CISTEINA

CERTIFICATO D'ANALISI
CERTIFICATE OF ANALYSIS

PRODOTTO PRODUCT — ***N - ACETIL- L-CISTEINA*** *(N.ACETYL-L-CYSTEINE)*

QUANTITA' — ***Kg. 5***

BATCH *IADF180307* — **COD.** *AC015*

CONF. *1 conf.da kg.5* — **SCAD.** *03.2021*

DESCRIPTION	WHITE CRYSTALS OR CRYSTALLINE POWDER
IDENTIFICATION	POSITIVE (IR)
SPECIFIC ROTATION	+ 25.30° (+21.3 / +27.0°)
PH	2.17 (2.0-2.8)
HEAVY METALS	< 10 ppm
As	< 0,5 ppm
Pb	< 1 ppm
Cd	< 0,5 ppm
Hg	< 0,1 ppm
IRON	< 20 ppm
ZINC	< 10 ppm
PARTICLE SIZE	80% PASS 20 MESH
TOTAL IMPURITIES	< 2,0%
TOTAL PLATE COUNT	CONFORM (<=1000 cfu/g)
YEAST AND MOULD	CONFORM (<=100 cfu/g)
E.COLI	NEGATIVE
SALMONELLA	NEGATIVE
LOSS ON DRYING	0,11%
RESIDUE ON IGNITION	0,05%
ASSAY	100,01% (98,5 – 101,0%)

09.2018

COPY OF THE ORIGINAL DOCUMENT, VALID ALTHOUGH NOT SIGNED.

NATUR-FARMA S.R.L.
Via Mazzini 29/F 37040 Pressana (VR)
C.F / P.Iva : 04022190237
info@naturfarma.it Tel.0442 411196

22/01/19

VIT. C

CERTIFICATE OF ANALYSIS

**Product Name:**	VITAMIN C (ASCORBIC ACID)
**CCL Product Code:**	P2213
Batch Number	6052331711179
Date of Manufacture	23/11/2017
Re-Test Date	22/11/2020
CAS No	50-81-7
Molecular Formula	$C_6H_8O_6$
EC/EINECS Number	200-066-2
Source	Fermented on Sorbitol Ex Corn (Vegetable)
Country of Origin	China
Compound Ingredient	None
Country of Manufacture	China
Suitable for Vegetarians	Suitable
Suitable for Vegans	Suitable
FEMAS (Feed Materials Assurance Scheme) Assured	Yes

Test	Specification	Result
Appearance	Crystals/Crystalline Powder	Conforms
Colour	White to off White	Conforms
Aroma	Characteristic	Conforms
Flavour	Characteristic	Conforms
Identification	Positive	Conforms
Assay	99.0%-100.5%	99.9%
Loss on Drying	Approximately 1%	0.04%
pH	2.1-2.6	2.5
Residual Solvents (Methanol)	Max 3,000ppm	Conforms
Lead (Pb)	Max 3ppm	<2.0ppm
Cadmium (Cd)	Max 1ppm	<1.0ppm
Arsenic (As)	Max 1ppm	<1.0ppm
Mercury (Hg)	Max 0.1ppm	<0.1ppm
Copper (Cu)	Max 5ppm	<5.0ppm
Iron (Fe)	Max 2ppm	<2.0ppm
Total Plate Count (TVC)	Max 1,000cfu/g	<1,000cfu/g
Yeast & Moulds	Max 100cfu/g	<100cfu/g
E.Coli	Max 10cfu/g	Negative
Salmonella	Negative	Negative

INFORMED SPORT Registered Site FEMAS

NATUR-FARMA S.R.L.
Via Mazzini 29/F 37040 Pressana (VR)
C.F / P.Iva : 04022190237
info@naturfarma.it Tel.0442 411196
22/01/19

Vit. C

GMO Status	Non-GMO	Non GMO
Irradiation Status	Non Irradiated	Non Irradiated
TSE/BSE Status	TSE/BSE Free	TSE/BSE Free

This material conforms to the BP Standard.

This material is to be stored in a tightly sealed bag/container and to be kept in a cool place away from moisture and direct sunlight.

Please note that surveillance testing may mean that not all the parameters stated on this specification are tested for every batch.

We confirm that the information above is sourced from the original manufacturers/suppliers Batch Certificate of Analysis.

The specifications detailed in this COA conform with Version 9 of our Technical Dossier for Vitamin C (Ascorbic Acid).

To be used as per local legislation.

Change History

Version	Change	Customer Notification required Yes / No
1	First Issue	N/A

Document Approval

Task	Role	Name	Sign	Date
Approved By	QC Technician	M. Sosnin	M. Sosnin	09/03/2018

NATUR-FARMA S.R.L.
Via Mazzini 29/F 37040 Pressana (VR)
C.F / P.Iva : 04022190237
info@naturfarma.it Tel.0442 411196

22/01/19

AC. ALFA LIPOICO

CERTIFICATE OF ANALYSIS

Product Name: **ALPHA LIPOIC ACID (DC Grade)**

CCL Product Code: **P34176**

Batch Number ALA.1406231

Molecular Formula $C_8H_{14}O_2S_2$

Test	Specification	Result
Appearance	Granular	Conforms
Colour	Yellowish	Conforms
Assay (by HPLC USP Method)	99.0% - 101.0%	99.5%
Melting Point	60 °C – 62 °C	62°C
Total Impurity	Max 0.5%	0.05%
Individual Impurities	Max 0.10%	0.04%
Particle size	20-60 Mesh	Complies
Loss on Drying	Max 0.5%	0.1%
Residue on Ignition	Max 0.10%	0.04%
Heavy Metals	Max 10ppm	<10ppm
Lead	Max 3ppm	<3ppm
Arsenic	Max 1ppm	<1ppm
Cadmium	Max 1ppm	<1ppm
Mercury	Max 0.1ppm	<0.1ppm
Cyclohexane	Max 2000ppm	614ppm
Toluene	Max 30ppm	Not Detectable
Ethyl acetate	Max 250ppm	42 ppm

success starts with the finest ingredients...

NATUR-FARMA S.R.L.
Via Mazzini 29/F 37040 Pressana (VR)
C.F / P.Iva : 04022190237
info@naturfarma.it Tel.0442 411196
22/01/19

Ac. ALFA LIPOICO

ALPHA LIPOIC ACID -Continued

**Test**	**Specification**	**Result**
Total Plate Count (TVC)	Max 1,000cfu/g	<10cfu/g
Yeast & Moulds	Max 100cfu/g	<10cfu/g
E.Coli	Absent/g	Not Detectable
Salmonella	Absent/g	Not Detectable
GMO Status	Non-GMO	Non-GMO
Irradiation Status	Non Irradiated	Non Irradiated

This material conforms to the USP

This material is to be stored in a tightly sealed bag/container and to be kept in a cool place away from moisture and direct sunlight.

We confirm that the information above is sourced from the original manufacturers/suppliers Batch Certificate of Analysis.

To be used as per local legislation

QA Approval

Signature:

Checked By: Jason Gibbons

INFORMED-SPORT Registered Site
INFORMED-CHOICE Trusted by sport
FEMAS
ORGANIC
success starts with the finest ingredients...
